# dCITE: Measuring Necessary Cladistic Information Can Help You Reduce Polytomy Artefacts in Trees

**DOI:** 10.1371/journal.pone.0166991

**Published:** 2016-11-29

**Authors:** Michael J. Wise

**Affiliations:** 1 Computer Science and Software Engineering, The University of Western Australia, Perth, Australia; 2 The Marshall Centre for Infectious Diseases Research and Training, The University of Western Australia, Perth, Australia; University of Lausanne, SWITZERLAND

## Abstract

Biologists regularly create phylogenetic trees to better understand the evolutionary origins of their species of interest, and often use genomes as their data source. However, as more and more incomplete genomes are published, in many cases it may not be possible to compute genome-based phylogenetic trees due to large gaps in the assembled sequences. In addition, comparison of complete genomes may not even be desirable due to the presence of horizontally acquired and homologous genes. A decision must therefore be made about which gene, or gene combinations, should be used to compute a tree. Deflated Cladistic Information based on Total Entropy (dCITE) is proposed as an easily computed metric for measuring the cladistic information in multiple sequence alignments representing a range of taxa, without the need to first compute the corresponding trees. dCITE scores can be used to rank candidate genes or decide whether input sequences provide insufficient cladistic information, making artefactual polytomies more likely. The dCITE method can be applied to protein, nucleotide or encoded phenotypic data, so can be used to select which data-type is most appropriate, given the choice. In a series of experiments the dCITE method was compared with related measures. Then, as a practical demonstration, the ideas developed in the paper were applied to a dataset representing species from the order Campylobacterales; trees based on sequence combinations, selected on the basis of their dCITE scores, were compared with a tree constructed to mimic Multi-Locus Sequence Typing (MLST) combinations of fragments. We see that the greater the dCITE score the more likely it is that the computed phylogenetic tree will be free of artefactual polytomies. Secondly, cladistic information saturates, beyond which little additional cladistic information can be obtained by adding additional sequences. Finally, sequences with high cladistic information produce more consistent trees for the same taxa.

## Introduction

### Data for building phylogenetic trees

Biological researchers create phylogenetic trees as hypotheses of the evolutionary relationships between species. Historically, tree building was based on phenotypic properties of the taxa; phenotypic information is still used, in whole or part, to build some trees. However, since the pioneering work of Woese on the use of the small subunit (SSU) rRNA gene to distinguish species [1], protein and nucleotide sequence data has been used as input for for numerous tree building programs. Over time, other genes have been proposed, e.g. the bacterial gene recA [2], because SSU rRNA may not provide sufficient variation to distinguish different species. However, it soon became clear that no single gene can be expected to resolve every set of taxa; see, for example Penny et al’s comment on “MUTOG, the Myth of a Universal Tree from One Gene” [3]. A movement began to base phylogenetic analyses on concatenated sequences. For example, a concatenation of 13 mitochondrial protein sequences and a second concatenation of 12S and 16S genes were used to reconstruct the evolutionary history of guinea-pigs [4]. A study of 14 yeast taxa [5] expanded the number of genes included in concatenations up to 106, with the aim of eliminating incongruence between the computed trees. Among other things, the study concluded that a single gene, or small set of genes, will be inadequate for unambiguously resolving phylogenetic histories. However, what the study made clear is that one may not need entire genomes to create dependable phylogenetic trees.

D’Erchia et al (1996) [4] called such concatenations of genes “super-genes”, but the term most frequently used for such concatenations is “super-matrix” (see the review de Queiroz & Gatesy (2006) [6]). The idea is that standard tree-building programs compute trees from the set of concatenations, much as they would for databases of single gene or protein sequences. It is now appreciated that each gene/protein in the concatenation may have its own evolutionary history (see, for example, the review Zhang & Yang (2015) [7]), which implies the need for different parameters, both for rate variables and for the proportions of invariant sites, across the concatenation. (Sites may be thought of as the columns in a multiple sequence alignment.) One way around this problem is to use a Bayesian framework; Different data can be combined, each with its own model in its own partition, e.g. protein sequences, nucleotide sequences, RNA sequences including information from RNA secondary structure and categorical/encoded phenotypic data [8, 9]. However, a major limitation is that only a small number of genes, proteins or other data sources, can be included due the heavy computational load, even on modern multi-core computers. In other words, it may not be feasible to include all genes and, from the Rokas et al [5] study, it may not be necessary. More importantly, given the ubiquity of horizontal gene transfer in prokaryotes [10], and even eukaryotes [11], using whole genomes or all genes concatenated runs the risk of including genes which have their origins in other species and can therefore create a discord between the computed gene tree and the actual species tree [12, 13], though one recent approach has been to use information about the presence or absence horizontally gene transfer, gene duplication and gene loss events to make inferences about speciation [14].

If not all genes/proteins in a genome/proteome are to be included, the issue, then, is to determine the most representative and the most informative set of genes or proteins upon which to base the task of computing a reliable species tree spanning the taxa of interest. The need for the most representative set is intuitively clear, and encompasses issues such as avoidance of paralogues (gene duplication/extinction) and horizontally acquired genes [12, 13], and minimisation of “nonphylogenetic signals”, such as nucleotide composition bias and mutational saturation at particular positions, often third codon positions [15, 16]. Artefacts due to inclusion of paralogues can be minimised by only using mono-copy genes, while inclusion of horizontally acquired genes can be made less likely by choosing genes that are highly expressed, on the basis that highly expressed genes evolve more slowly [13, 17] and are more resistant to horizontal transfer [18]. Issues arising from biases in nucleotide composition and mutational saturation at particular sites can be ameliorated by using protein, rather than nucleotide, sequence data for protein-coding genes [15]. In addition, differences in computed trees, resulting from differences in methods for the input multiple sequence alignments, may be minimised when protein sequence data are used [19]. That said, rates of evolution can vary considerably between species—see, for example [20]—and analysis based on nucleotide sequences may be more appropriate for protein coding genes from very slowly evolving species. Finally, there is the important issue of taxon sampling, for which no simple solution exists starting with the need to adequately sample the space of taxa [21], with Townsend & López-Giráldez (2010) [22] highlighting the need to target strategically the deepest in-group taxa, i.e. those closest to the time of speciation. The literature, and attendant controversies, regarding taxon selection has been extensive [23].

### Quantifying the information content of trees

Beyond the need to put together the most representative set of taxa is the need for the data characterising the set to be as informative as possible, which has also been known for some time [3]. In particular, we need to determine the information content of the proposed set of data representing taxa to decide if there is at least the minimum phylogenetic information, below which it will be impossible to construct as fully resolved phylogenetic tree as the input taxa allow. This amount of information I shall call *necessary cladistic information*. For example, based on results from phylogenetic tree building based on 2,538 RuBisCo large subunit *rbcL* genes, each 1,428bp, Källersjö et al [24] argues for the inclusion in phylogenetic analyses of information from the highly variable third codon position, despite the fact that the third position data are saturated. The paper finds that, when only the more conserved first and second codon positions are used, 431 of the taxa can be resolved, but this rises to 1,400 when all three positions are used. Another way of viewing these findings is that adding information from the third codon position has considerably added to the cladistic information, allowing the phylogenetic relationships of more taxa to be resolved.

A series of papers starting with Nelson & Platnick (1981) [25] have canvassed the information content of “classifications”, i.e. phylogenetic trees, and different ways to measure it. Wortley & Scotland (2006) [26] calls them “measures of potential utility”. Measures of cladistic information offer another way of looking at the question of how good a particular tree is, in addition to comparisons with the “true tree”, where this is known a priori, or can be inferred. Two basic approaches have emerged: measurements on computed trees and measurements on the multiple-sequence alignments (or other data) that are inputs to the tree building process. The earliest approaches were on trees. Maddison (1989) [27] distinguishes between “hard” and “soft” polytomies, where the former are due to multiple speciation events having occurred in a common timeframe, while the latter are due to ambiguities in tree construction. All the authors looking at cladistic information encoded in phylogenetic trees have, explicitly or implicitly, made use of the assumption that, unless there is evidence to the contrary, all polytomies are soft polytomies. With this in mind, Thorley et al (1998) [28] defines the phylogenetic (or cladistic) information content of a tree or subtree to be

$$-log(\frac{number\;of\;permitted\;trees}{number\;of\;possible\;trees})$$

A binary (sub) tree permits only 1 tree. Thorley [29] provides a comparison between cladistic information content as defined above, and metrics proposed in earlier studies. Taking a different approach, the EDIBLE program computes the Fisher information, or expected information from an input phylogenetic tree, allowing different topologies and branch lengths to be compared [30]. EDIBLE was used to evaluate the phylogenetic information of different genes, and gene combinations [31]. In particular, phylogenetic trees were computed and their underlying models were optimised before the trees were passed to EDIBLE. The tree-based phylogenetic information was then used to compare the input sequences [31].

### Quantifying the information content of the alignments used to build trees

The problem with tree-based metrics is that they necessarily involve first creating a tree from which the information content can be derived. However, the information content found in the output tree is implicit in the input data used to create the tree, although the relationship will not be linear with the number of taxa because the number of possible trees increases factorially. If based on the input data, cladistic information is about characters rather than trees, so it is worthwhile to distinguish between *input cladistic information* and *output cladistic information*.

A number of input cladistic information metrics have been proposed:

- Δ_*min*_ [26], which the authors characterise as “the minimum number of parsimony informative character-state changes”. For a given site, this is defined to be the number of different character states minus 1, where each state change must be present in at least two taxa. (The character states are set of values seen at a given site.) The site scores are then summed across all the sites.
- Total Cladistic Information Content (Total CIC) [32]. Total CIC sums the function *I*(*χ*), found in section 9.3 of Steel & Penny [33], across all characters/sites. *I*(*χ*) quantifies the information to be found in a single r-state character as the number of trees where a site can be explained without homoplasy. Assuming a particular character/site partitions into *r* states with counts of instances labelled *a*_1_, *a*_2_, …, *a*_*r*_, then
  $$I\left(\chi \right)=\sum_{j=3}^{n-r+1}\left(1-b_{j}\right)log\left(2j-3\right)$$
  where *b*_*j*_ = |{*i* : *a*_*i*_ ≥ *j*}| and *n* is the number of taxa. The variable *b*_*j*_ represents, for a given count *j* of instances of a given character state, the number of states with that count or greater. For example, in an alignment of 50S ribosomal protein L11 sequences representing 10 taxa, in which one site has 7 instances of G and 3 of A, then *b*_3_ = 2 and *b*_4_ = *b*_5_ = *b*_6_ = *b*_7_ = 1.

Δ_*min*_ captures the notion, from parsimony, that there must be different character states in a site for there to be cladistically useful information, and counting the states relates to the potential for splits between clades, and between species, in the final tree. On the other hand, Total CIC relates the utility of characters to numbers of permitted trees.

Both Δ_*min*_ and Total CIC capture many intuitions about the information, encoded in a multiple sequence alignment, that will be used to create a phylogenetic tree. However, neither is ideal. The shortcoming of Δ_*min*_ is that each character state counts equally, so long as it appears more than the threshold number of times. However, the utility of a character, say with 2 states, will be very different in the case where the split in counts between the states is *n*/2, *n*/2 − *n* the number of taxa—versus when the split is 2, *n* − 2. (This follows from the definition of CIC above. Both the number of possible trees and the number of permitted trees are, in the limit, *O*(*n*!). Assuming that a split between two clades occurs due to information at a given site/character, CIC is maximal when the split is even.) Total CIC is able to deal with this issue, but in Total CIC the utility of a character rises, without limit, with increasing count of taxa (approximately *n* × *log*(*n*)), because *b*_*j*_ = 0 in the above equation for all *j* greater than the count of the most numerous state, so *b*_8_ = *b*_9_ = *b*_10_ = 0 in the above example. Such unconstrained growth is an undesirable model for the underlying information because, given a fixed number of characters (in practice fixed sequence length), and fixed alphabet size, the number of possible unique sequences is limited, particularly given the highly conserved genes/proteins one typically uses for phylogenetic reconstructions, where many sites are likely to be invariant.

## Methods and Databases

### A New-Old Method for Computing Cladistic Information

Building from the predecessor methods, the following method, called “deflated Cladistic Information (based on) Total Entropy” (or dCITE) is proposed. The method sums the entropy scores for each character/site, only counting character states that have at least 2 instances. That is:

$$CITE=-\sum_{i=1}^{n}\sum_{j=1}^{m_{i}}p_{j}\times log_{2}\left(p_{j}\right)$$

where *m*_*i*_ is the number of character states, present in at least 2 taxa, at site *i*, and *p*_*j*_ is the proportion of the *j*^*th*^ character state at site *i*. Below is a small example to illustrate the method. To keep the example compact, assume that the parsimony assumption above is not made; characters with a count of 1 will be included in the computation.

Site 12345

Tax1 abaca

Tax2 abaca

Tax3 aaaac

Tax4 aacad

The corresponding CITE score is 0 + 1 + 0.8113 + 1 + 1.5 = 4.3113.

The raw CITE score is then deflated in two ways to produce a *dCITE* score. Firstly, sequences that are completely identical to other sequences do not contribute additional cladistic information; on the evidence of these sequences the taxa are indistinguishable. However, duplicate sequences will alter scores because percentages of characters for a given site will be increased in the duplicated taxa at the expense of characters from unique taxa. Tax2 is duplicate of Tax1 in the above example. For that reason, from a set of identical taxa, one is retained and the rest are ignored. (Ignored taxa are, however, reported by the software so the user is made aware of the duplication.) Similarly, a site, whose states recapitulate the splits at another site, is ignored. In the example above, Site 2 and Site 4 have the same split: Tax1 with Tax 2, and Tax 3 with Tax4. Site 4 adds no new information beyond that provided by Site 2, so the contribution to the cladistic information from Site 4, and therefore to the correspond CITE score, can be ignored. (However, Site 4 does provide confirmation of the split seen in Site 2 so, depending on the tree building algorithm, could add weight to the cladistic information provided by Site 2.) This sort of duplication could occur, for example, in RNA data when secondary structure is formed and there is base-pairing across stems; in a stem, the base on one side will determine the base on the other side. Such patterns are also characteristic of haplotypes, leading to the concept of tag SNPs in the HapMap project [34]. Returning to the example above, the dCITE score is now 0 + 0.9183 + 0.9183 + 0 + 1.5850 = 3.4216 (from 3 taxa). As a more realistic example, in a set of 4 unique ATPA—ATP synthase alpha chain—proteins (average length 525aa), there were 32 informative sites with total CITE score of 32. However, the dCITE score was 3 from just 3 sites with different split patterns.

dCITE retains the notion of summing the contribution of each character, but those contributions are now weighted, with the greatest weights occurring when the counts of character states are balanced at a given site. In addition, as is evident from Site 5, *n* different character states at a given site will return higher scores than comparable sites with *n* − 1 character states. This is reasonable when one considers the increase in information available from an *n*-way split versus an *n* − 1-way split. However, while dCITE has the desirable property that invariant sites do not contribute to the score, a worst-case exists where every character state in the alphabet appears in approximately equal numbers at a given site. That site would convey little cladistic information but the dCITE score will be close to maximal. Site 5 in the example above has that property. The problem is excess diversity of characters at a particular site, and is related to the problem of mutational saturation [16], except that the latter is an average property across all sites, or all instances of a class of sites, e.g. all third codon positions, while the former is site specific. The impact of excess diversity was tested in Experiment 1, described below.

### Experiment 1

Three experiments were undertaken to examine the application of dCITE in a number of settings. The first experiment sought to compare dCITE with tree-based CIC and multiple sequence alignment based Total CIC and Δ_*min*_. Three sets of evolutionarily highly conserved sequences were obtained: ATPA, ATP synthase alpha chain, TYMS, thymidylate synthase and RL11, 50S ribosomal protein L11. For each of these proteins, sets of homologues were obtained using BLAST sequence search [35] on the UniProt web site. For each set, species diversity was enhanced by only accepting one sequence with a given species suffix in the sequence identifier. In addition, the sets were sieved using the Ucluster function from the Usearch suite [36], so that near duplicates (greater than or equal to 98% identity) were removed, yielding 603, 286 and 520 sequences, respectively. For each database, starting with 4 randomly chosen sequences, a multiple-sequence alignment was computed using Muscle [37]. From the multiple-sequence alignments 100 bootstrapped phylogenetic trees were created using a multi-threaded version of the maximum-likelihood tree builder Phyml [38]. Then, an additional randomly chosen sequence was added and the process repeated, and so on, until the final set contained 200 sequences. When the 197 sets of bootstrapped trees were completed a number of metrics were computed.

For each of the consensus forming rules—Strict, Majority Rule (MR, i.e. 50th percentile) and Majority Rule extended (MRE)—the Consense application from the Phylip suite [39] was used to build a consensus tree for each of the sets of trees. Then, for each of the consensus trees, a Python application computed the tree based CIC statistic, the minimum bootstrap count for the clades in the tree and the *degeneracy* of the tree. Degeneracy is a measure of the number and degree of polytomies contained in the tree. That is, using a recursive algorithm, degeneracy is the sum across all levels of a tree *t* and all subtrees *t*_*i*_, of differences between the actual degrees of subtrees (i.e. number of branching points) and the ideal (a dichotomy).

$$degeneracy\left(t\right)=\{\begin{array}{ll} 0, & \text{if t is a leaf}.\\ degree\left(t\right)-2+\overset{degree\left(t\right)}{\underset{i=1}{\sum }}degeneracy\left(t_{i}\right), & \text{otherwise}. \end{array}$$

Degeneracy represents a refinement of Colless’ Consensus Fork Index [40], which counts the number of internal nodes. (In this study, the minimum degeneracy score was 1, rather than 0, because Consense structures the top-level node as a trifurcation.)

A second Python application was used to compute Δ_*min*_, Total CIC, CITE and dCITE from the multiple sequence alignments that were used to create the trees. Because dCITE involves deletion of duplicate sequences prior to computing the metric, this was done for all the analyses as duplicate-deletion affects the count of taxa. In addition, while dCITE is able to treat the gap character as a fifth (nucleotide) or twenty-first (protein) state, this was ignored for these experiments.

A final Python application was used to calculate character diversity statistics for each of the input multiple sequence alignments. The character diversity at a given, variable site is defined as the number of character states represented at that site divided by the minimum of the number of non-gap characters found at that site or the alphabet size. In other words, character diversity measures the extent to which the entire alphabet appears at a given site, constrained by the number of taxa. The mean, median and maximum character diversity values were then calculated, together with the fraction of values less than or equal to 0.5.

### Experiment 2

The second experiment sought to examine the impact on the quality of the computed phylogenetic trees of diminishing cladistic information from a single, synthetic dataset. The starting point for this experiment was the ATPA dataset containing 50 taxa retained from Experiment 1. A starting tree was computed using MrBayes [41] (WAG amino acid rate matrix, variation between sites being modelled with a gamma function and a percentage of invariant sites—invgamma—and a birth-death clock model), based on a multiple sequence alignment created using Muscle [37]. (A maximum-likelihood tree could equally have been used.) Armed with the starting tree, and taking one of the 50 sequences as the root (i.e. ancestral) sequence, Pyvolve [42] was used to create a set of 50 gap-free, evolutionarily related, synthetic sequences (WAG substitution matrix). In addition, a percentage of randomly chosen sites were made invariant, the number being equal to the percentage of invariant sites in the source ATPA dataset. Using settings similar to those used for the starting tree, a Bayesian “True Tree” was computed for the synthetic dataset.

A Muscle-generate multiple sequence alignment of the synthetic dataset was used in the following way. As in Experiment 1, 100 bootstrapped phylogenetic trees were computed using Phyml, with a Majority Rule Extended consensus tree computed using Consense. The application TreeCmp [43] was then used to compare the consensus tree against the True Tree across four metrics: Robinson-Foulds Distance, Estabrook’s Quartet Distance, Steel and Penny’s Path Difference Distance and the authors’ own metric, Matching Split Distance. The dCITE score for the input multiple sequence alignment and degeneracy of the consensus trees were also recorded. Then, ten randomly chosen amino acids were removed from the multiple sequence alignment, and the process of computing a new tree and measuring the difference with the True Tree was iterated until a minimum number of sites (20) remained.

### Experiment 3

The third experiment puts together many of the ideas that have been developed in this paper, in a comparison of three phylogenetic trees examining the evolution of *Helicobacter pylori* in the context of other members of the order Campylobacterales. Campylobacterales includes the family Campylobacteraceae, which includes the genera *Campylobacter* [44] and *Arcobacter* [45], and the family Helicobacteraceae, which includes the genera *Helicobacter* [46] and *Wolinella* [47]. The list of species included in the study can be found in Table B in S1 File. If we accept the preference for single copy genes (at least at the protein level), a preference for avoiding horizontally transferred genes, and also the argument that highly expressed genes are less likely to have been acquired horizontally, the following method was used to derive such a set of protein sequences from which to build phylogenetic trees. While RNA expression data does exist for *Helicobacter pylori* on services such as ArrayExpress (at EBI) and GEO (at NCBI), each experiment has involved different custom-made arrays and they are therefore not comparable. By contrast, there is a considerable body of data for *Escherichia coli*. The starting point, therefore, was a list of highly expressed *E. coli* proteins, taken from proteomic data [48]. High mRNA expression, across a range of conditions, was confirmed using the Genevestigator database https://www.genevestigator.com/. Confirmation was also sought for the proteins’ presence in *Helicobacter pylori*. Unsurprisingly, the resulting list, which can be found as Table C in S1 File, is dominated by proteins involved in the transcription and translation machinery. In addition to the highly expressed genes/proteins, two moderately expressed proteins were added to the database: mutY, A/G-specific adenine glycosylase, and trpC, bifunctional indole-3-glycerol phosphate synthase/phosphoribosylanthranilate isomerase. (It is the indole-3-glycerol phosphate synthase moiety that is of interest here.) The reason for including the two additional sequences is to allow comparison with the Multilocus Sequence Typing (MLST) technique [49], which is the current state of the art in strain identification, particularly for *H. pylori*. MLST involves strain identification through the concatenation of nucleotide or protein sequence fragments obtained using organism-specific sets of primers for a range of genes. The genes surveyed, and the locations of the primer sites, vary from species to species.

As in Experiment 1, a multiple sequence alignment was computed using Muscle [37]. Having assembled the database of 87 sets of protein sequences, the dCITE method was run on each set and on each pairwise combination of sets. In some cases, target sequences were simply not found for specific strains, likely due to sequence assembly or gene-calling errors, because these are all single copy, essential proteins. Taking in to account the availability of the corresponding sequences, two pairs of proteins were selected as being the most informative: glyS+pheT (dCITE score of 1648.40 bits across 1,230 informative sites, out of 1,461 sites overall) and lon+rpsA (1059.76 bits from 960 informative sites out of 1,360 sites overall).

BIGSdb is a database of MLST sequence data for a range of species [50]. In particular, data from a large number of isolates is available for both *Campylobacter jejuni* and *H. pylori*, and data for 6 of the 7 protein fragments available for *H. pylori* overlap protein fragments from *C. jejuni*: atpA, efp, mutY, ppa, trpC and engA. (The protein known as yphC in the *H. pylori* BigsDB dataset, is more widely known as engA or der.) To create an equivalent concatenation, starting with the complete protein sequences used in this study, sample concatenations of the 6 protein fragments from *H. pylori* and *C. jejuni* were downloaded from BIGSdb and then aligned with concatenations of the corresponding sequences from this study. (The MLST sequences are much shorter.) The multiple-sequence alignment was then edited using JalView [51], leaving only the portions that correspond to the MLST data. The atpA+efp+mutY+ppa+trpC+engA concatenation corresponding to the MLST data had a dCITE score of 480.85 from 467 informative sites out of a starting count of 934 sites. I shall call this concatenation MLST.

For each of the 3 concatenations, phylogenetic trees were computed using MrBayes [41]. In particular, a partitioned analysis was used, with each protein occupying its own partition. An invgamma model was used to model both the per site evolutionary rate and the percentage of invariant sites, with both the alpha parameter of the gamma distribution (the shape parameter) and the proportion of invariant sites being allowed to vary between partitions. The computation was stopped when the average standard deviation of split frequencies was less than 0.01. As with Experiment 2, TreeCmp [43] was used to compare pairs of trees across the four metrics: Robinson-Foulds Distance, Estabrook’s Quartet Distance, Steel and Penny’s Path Difference Distance and the authors’ own metric, Matching Split Distance.

## Results

### Experiment 1

The discussion here will focus on the ATPA dataset; corresponding Figures for the TYMS and RL11 datasets may be found as Figs A-D in S1 File. The figures have been produced using Version 3.1 of the R suite of mathematical/statistical functions [52].

**Fig 1:**
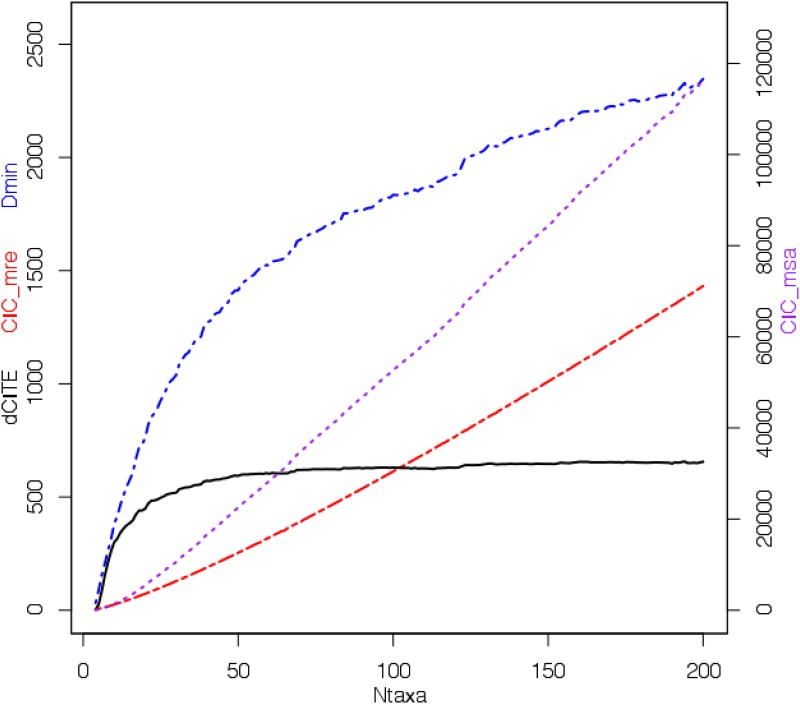
Experiment 1. Plot of dCITE score, Δ_*m*_*in*, CIC and TOTAL CIC For the ATPA-98 dataset, the plot of dCITE score (solid black line), Δ_*m*_*in* (shown as a blue . — . — line), computed from the input multiple sequence alignments, and CIC scores based on the MRE trees (shown in red using a . — . — line) are plotted versus the counts of taxa. TOTAL CIC scores based on the input multiple sequence alignments, also plotted against counts of taxa, are shown using purple dashed lines. Note the different Y-axis for these scores, on the right hand side.

Fig 1 shows scores for the different metrics based on the 197 input multiple sequence alignments for the ATPA dataset: dCITE (shown as a black, solid line), Δ_*min*_ (blue, dot-dash line) and TOTAL CIC (CIC_msa, purple dashed lined), or the 197 tree-based CIC scores (shown in red), which is for the set of MRE trees. (The graph for CIC values of the MR and Strict agreement trees will be lower as these will have many more polytomies.) Note that CIC_msa is drawn to a much larger scale (on the right hand side). The point to note here is that, after initially rising, dCITE scores plateau, which implies that the amount of cladistic information in the multiple sequence alignment has, essentially, saturated, implying that adding more taxa adds little further cladistic information. This is further explored in Experiment 2. (The possible confounding effect of mutational saturation—as approximated here by character diversity—is discussed below.) Δ_*min*_ may also be levelling off, but the two CIC based scores continue to rise steadily.

**Fig 2:**
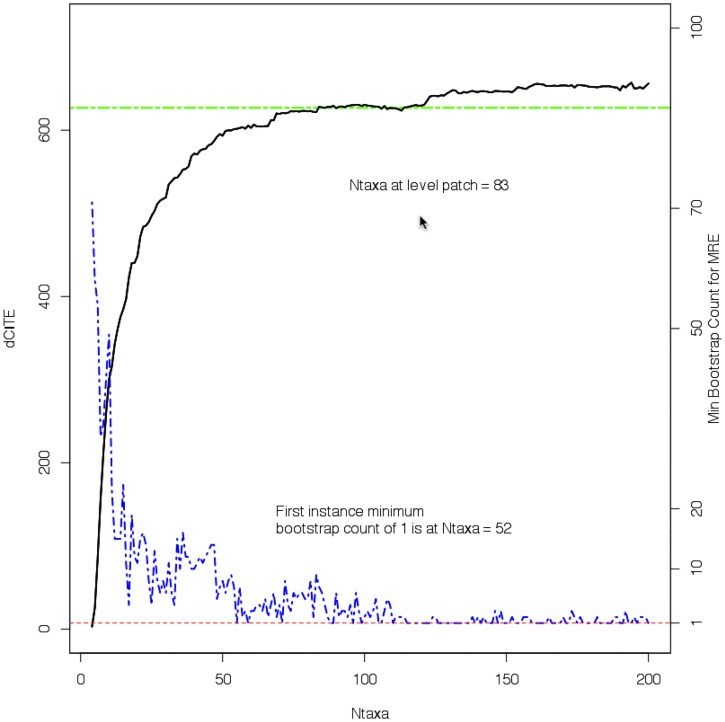
Experiment 1. Plot of dCITE score, minimum bootstrap count and model of the distribution. For the ATPA-98 dataset, the plot of dCITE score (black solid line) and Minimum Bootstrap Count (for trees created using the Majority Rule Extended consensus rule, shown as a blue . — . — line) versus the counts of taxa. A horizontal green line marks the first “level patch”, i.e. where the sum of the differences between adjacent dCITE scores over 10 counts of taxa is no more than 15.

Fig 2 shows (in black) the dCITE scores for each of the 197 multiple sequence alignments for the ATPA dataset. As noted in Fig 1, after initially rising, the dCITE scores plateau. More pragmatically, the count of taxa where the dCITE scores level off, $\bar{N}$, can be estimated by looking for the first count where, for the next 15 counts, the sums of differences between scores from adjacent counts is less than or equal to 15. That is:

$$\bar{N},\sum_{j=\bar{N}}^{\bar{N}+15}|dCITE_{j+1}-dCITE_{j}|\leq 15$$

This is shown in green, and a legend highlights the count where this has occurred. Also shown, in blue, is the plot of the minimum bootstrap counts for each set of taxa, against the count of taxa, for the consensus trees computed by Consense using the Majority Rule Extended (MRE) algorithm [39]. The ultimate points in the MRE algorithm are where a clade is supported by just the one tree, i.e. the clade has no other support bar itself (but is not contradicted by any other clade prediction). The first such case for the ATPA dataset occurs for the dataset containing 52 taxa.

**Fig 3:**
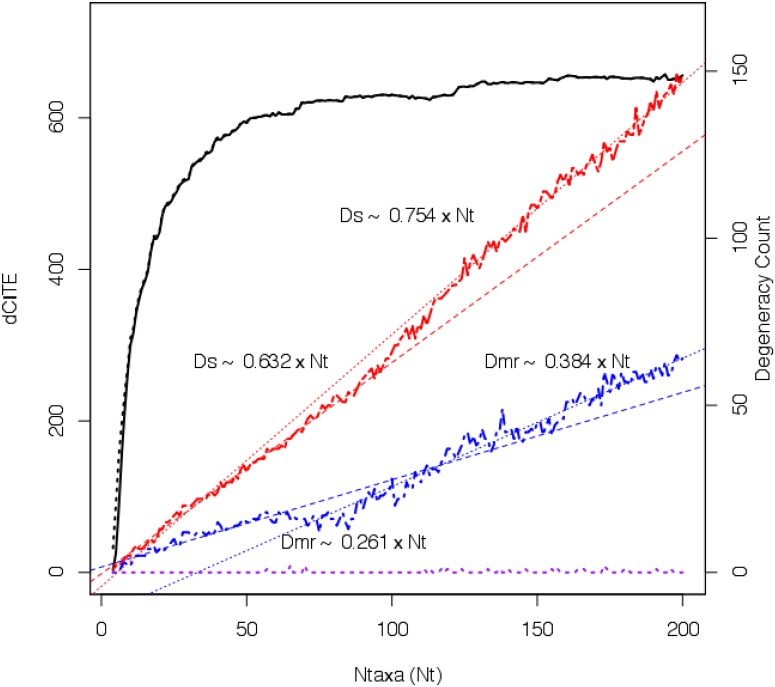
Experiment 1. Plot of dCITE score and degeneracy of corresponding trees. For the ATPA-98 dataset, the plot of dCITE score (solid black line) plotted against counts of taxa. The dashed black line next to the dCITE score graph is the graph of the corresponding CITE scores. Based on the Y axis on the right hand side there are the plots of degeneracy scores for Strict consensus trees (shown in red in red using a . — . — line), Majority Rule consensus trees (shown as a blue . — . — line) and Majority Rule Extended consensus trees (shown shown as a - - - - purple line at the bottom of the graph). For the Strict consensus trees and the MR trees linear models (dashed red and blue lines, respectively) are also shown. For each of the linear models, the slope Degeneracy (D) with the counts of taxa (Ntaxa) is also noted.

In Fig 3, dCITE scores are plotted in black against *Ntaxa*, with y-axis on the left. Shown in red, with y-axis on the right, is a plot of the degeneracy scores for the set of Strict consensus trees *D*_*s*_. Two linear models, depicted using red dashed lines, were computed for the set of degeneracy scores. The first, encompassing the initial 65 data points has a slope of 0.632 (adjusted *R*^2^ = 0.992), while the second, taking in the final 65 data points has a slope of 0.754 (adjusted *R*^2^ = 0.975). Degeneracy scores for the Majority Rule consensus trees *D*_*mr*_ are shown in blue, together with the corresponding linear models. In this case the model for the initial data points had slope 0.261 (Adjusted *R*^2^ = 0.0.909) rising to 0.384 (Adjusted *R*^2^ = 0.887) for the final data points. By contrast, the degeneracy scores for the MRE trees are mostly 0, corresponding to dichotomies at every ancestral node, though, of course, the level of support can be very low (see Fig 1). A small number of polytomies are present for larger counts of taxa.

The slopes of the corresponding linear models for the TYMS and RL11 data sets are steeper than for the ATPA data (see Fig C in S1 File). This is likely due to TYMS sequences being approximately half the length of ATPA (mean length of 279aa versus 514aa), and RL11 being smaller still (mean length 144aa). The final thing to note in Fig 3 is the dashed black line, corresponding to CITE scores, that closely follows the dCITE curve. The line is most evident near the origin, indicating that the deflation of CITE scores for duplicate sites is most relevant for small counts of taxa, but even then the impact is generally very small (less than 1% difference for more than 15 taxa in the ATPA dataset).

The character diversity statistics for the ATPA dataset can be found as Table A in S1 File. For small sets of taxa, character diversity is dictated by the parsimony assumption; for 4 taxa each character state must appear twice so the diversity was 0.5, while the fraction of low values (less than or equal to 0.5) was 1.0. However, by the time there were 20 taxa (the size of the amino acid alphabet), the median diversity value was 0.15 while the fraction of low values was still 1.0. In the case of 200 taxa, the median diversity value had risen to 0.300, and the fraction of low values had dropped to 0.818, while the greatest diversity value for a single site was 0.8. The values for the TYMS and RL11 datasets closely followed those for the ATPA dataset. To gauge the impact of limiting character diversity to 0.75 at a given site (corresponding to 3 out 4 possible nucleotides), with no limit the dCITE score for 200 taxa in the ATPA data was 656.13; with a diversity cap in place the score fell to 642.11 (a difference of 2.1%).

### Experiment 2

**Fig 4:**
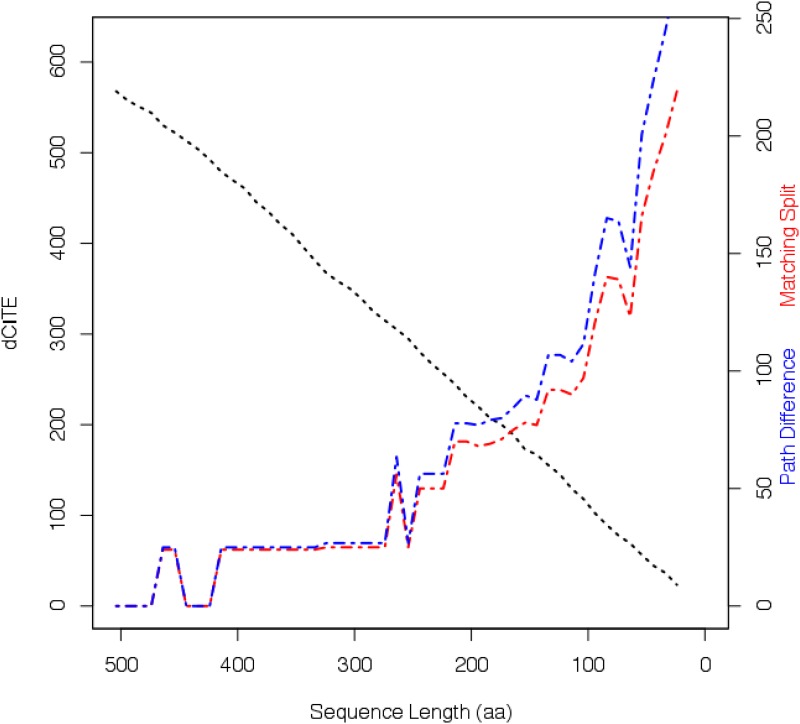
Experiment 2. dCITE score for input multiple sequence alignment, and Matching Split and Path Difference scores for computed consensus tree versus True Tee, plotted against sequence length. For the 50 taxon ATPA-98 dataset, the plot of dCITE score (solid black line) plotted against length of the input multiple sequence alignment. Also plotted against the length of the multiple sequence alignment are the Path Difference (blue . — line) and Matching Split (red . — line) scores for the difference between the Majority Rule Extended consensus trees and the True Tree.

Fig 4 is a plot of Path Difference scores (shown in blue) and Matching Split scores (shown in red), together with dCITE scores (shown in black), all compared with diminishing sequence length. Robinson-Foulds Distance and Quartet Distance are not shown; Robinson-Foulds Distance is relatively insensitive, while Quartet Distances produces very large values, though both measures tell essentially the same story as the other two measures. Each of the distance metrics should be 0 for identical trees, and minimal when comparing very similar trees. In this case, the metrics are minimal when many of the original amino acid sites remained and dCITE scores were high, but as the number sites declined below a threshold the level of dissimilarity with the True Tree increased markedly. What is also not shown in Fig 4 is the fact that, under Majority Rule Extended consensus method, the degeneracy of the consensus tree was 0 for all the sequence sets, apart from the last, 24aa set, when the degeneracy climbed to 2. This is in keeping with what was seen in Experiment 1. (Results for a second synthetic dataset based on the ATPA tree can be found as Fig D in S1 File.)

### Experiment 3

For each of the partitioned analyses—glyS+pher, lon+rpsA and MLST—two consensus trees were computed using MrBayes: an Allcompat tree, equivalent to Majority Rule Extended, and a Halfcompat tree, equivalent to using the Majority Rule. Table 1 lists, for the 6 combinations of sequence set and consensus-forming rule, the number of leaves in the tree, the tree CIC and the degeneracy of the tree. The dCITE, Δ_*min*_ and TotalCIC scores for the underlying multiple sequence alignments are also listed. What is worth noting here is that, while degeneracy scores of 1 are to be expected for Majority Rule Extended, the greater the cladistic information, by whichever measure, the less the degeneracy under Majority Rule and the smaller the drop in tree CIC scores from the MRE tree to the MR tree.

**Table 1:** Tree degeneracies, CIC scores, dCITE, Δ_*min*_ and TotalCIC scores for different sequence combinations and consensus tree forming rules.

Tree	Leaves	CIC	Degeneracy	dCITE	Dmin	TotalCIC
MLST_MRE	64	347.6	1	480.85	1064	24570
MLST_MR	64	320	14			
lon+rpsA_MRE	77	440.1	1	1059.76	2546	67378
lon+rpsA_MR	77	427.5	8			
glyS+pheT_MRE	76	432.9	1	1648.4	4272	98162
glyS+pheT_MR	76	427.4	4			

Table 2 comes in two parts. In the top three rows, for each sequence set the distance is computed between the Majority Rule Extended tree and the Majority Rule tree, with the ideal being that each of the distance scores should be 0 (indicating no difference). The results mirror the results observed in Table 1, where the greater the level of cladistic information the smaller the difference between the MRE tree and the MR tree. (The different tree metrics are entirely consistent on this.) In the lower 6 rows, MR and MRE trees computed from the different sequence sets are compared using TreeCmp. TreeCmp is able to deal with trees with overlapping sets of taxa by facilitating pruning of nodes and branches that are unique to one or other input tree. The comparisons show that the trees computed by the two pairs of sequences are more compatible than the either of those tree when compared with the MLST tree. The difference is even larger when one takes into account the larger number of nodes (and therefore branches) shared by the glyS+pheT and lon+rpsA trees, so the larger number of ways they could differ. Snapshots of the three MR trees, visualised using FigTree http://tree.bio.ed.ac.uk/software/figtree/ can be found as Figs E-G in S1 File.

**Table 2:** Distances between pairs of trees based on Robinson-Faults Distance (R-F), Bogdanowicz et al Matching Split Distance (M-S), Steel and Penny’s Path Difference Distance (P-D) and Estabrook’s Quartet Distance.

Tree1	Tree2	Ntaxa	Distances				dCITE
			R-F	M-S	P-D	Quartet	
glyS+pheT_MRE	glyS+pheT_MR	76	1.5	12	38.16	1,461	1,648.40
lon+rpsA_MRE	lon+rpsA_MR	77	3.5	33	71.87	15,557	1,059.76
MLST_MRE	MLST_MR	64	6.5	109	140.87	17,001	480.85
glyS+pheT_MR	lon+rpsA_MR	75	32.5	165	162.55	91,948	
glyS+pheT_MRE	lon+rpsA_MRE	75	35	178	192.13	87,314	
lon+rpsA_MR	MLST_MR	63	35	197	202.29	115,381	
glyS+pheT_MR	MLST_MR	64	32.5	202	224.97	115,483	
lon+rpsA_MRE	MLST_MRE	63	43	214	202.50	109,037	
glyS+pheT_MRE	MLST_MRE	64	39	215	230.17	110,230	

## Discussion

Deflated Cladistic Information based on Total Entropy (dCITE) provides an easily computed metric based that can be used to decide which combination of genes provides the most cladistic information to enable a robust phylogenetic tree to be computed. This minimum cladistic information has been called necessary cladistic information, and can be estimated by progressively adding taxa and finding the count where the the dCITE scores level off. Trying to resolve more than that number of taxa will be more likely to result in polytomies, as evidenced by the increased rate of degeneracy seen in the last tranche of taxa sets versus the first tranche (Fig 3 and Fig C in S1 File). This saturation of cladistic information also underlies the results of Experiment 2. With the full-length alignment, there was ample cladistic information so there were few polytomies. However, as sites were deleted there were, initially, few differences with the True Tree, suggesting that the remaining sites were, in effect, buffering the cladistic information, e.g. via sites with duplicated splits. Eventually, that redundancy was depleted and the number of polytomies begins to rise steeply. In other words, whether one views the phenomenon from the point of view of adding further taxa or deleting sites, polytomies result when there is insufficient cladistic information to distinguish the set of taxa.

Secondly, while the trade-off between freedom from polytomies and level of support for the different clades is well known, what this study shows is that greater cladistic information, as evidenced by greater dCITE scores (or the other metrics), results in lower degeneracy, i.e. fewer polytomies, for a given level of support, e.g. Majority Rule. The study also indicates that after an initial period, degeneracy scores rise approximately linearly with increasing numbers of taxa; comparison of the ATPA, TYMS and RL11 datasets also suggests that the lower the dCITE score the greater the rate of increase in degeneracy with added taxa (closer to a slope of 1, at which point every additional sequence causes an additional or an expanded polytomy). Thirdly, the results from the second experiment suggest that, even though the Majority Rule Extended consensus method produces trees that are free from degeneracy (i.e. polytomies), reflecting the low dCITE scores, the trees are likely to be significantly different from the True Trees. In summary, taken together, Experiments 1 and 2 suggest that low dCITE scores computed from input data indicate a shortage of cladistic information and will result in computed trees containing artefacts.

The applications of the dCITE method discussed in this paper have focused on protein sequence data. However, the dCITE method can be equally well applied to nucleotide or encoded phenotypic data. As mentioned in the Introduction, for protein-coding nucleotide data, while the protein translations may often be preferred, there are circumstances where nucleotide based methods may be more appropriate, e.g. for genes from slowly evolving or recently diverged species. However, the dCITE method can be used to inform the decision about which data type to use. The results suggest that the potential weakness in the dCITE method identified earlier—sites containing the entire alphabet in approximately equal proportions—does not occur (some character states are not represented). More importantly, the number of sites containing more than 75% of the alphabet is very low so their impact will be limited. Finally, now that the cost of genome sequencing is comparable to use of MLST primers followed by fragment sequencing, the results from Experiment 2 suggest that it is appropriate to use a method, such as dCITE, to find more informative sequence combinations than MLST upon which to base phylogenetic tree computations.

## Conclusions

The paper has discussed Deflated Cladistic Information based on Total Entropy (dCITE), a novel reworking of previously described metrics for computing the cladistic information content in the multiple sequence alignments that will be used to create phylogenetic trees. The method does not attempt to provide guidance about which taxa are to be included in the phylogenetic reconstruction. Rather, dCITE aims to provide guidance about whether there is sufficient information for a robust tree to be computed, i.e. a tree without artefactual polytomies. The dCITE method requires as input nothing more than a multiple sequence alignment; In particular, the method does not require *a priori* knowledge of the phylogenetic relationships between the taxa (or at least a subset of the taxa).

The first conclusion is that the greater the dCITE score the greater the likelihood of computing a robust, well-supported tree. Secondly, cladistic information saturates, beyond which little additional cladistic information can be obtained by adding additional sequences. Mutational saturation, where present, can be modelled by excess character diversity and can be excluded from dCITE scores. The experiments reported here suggest that, for a given set of taxa, data sets with sufficient cladistic information from highly expressed genes are likely to produce comparable phylogenetic trees. Finally, now that the cost of genome sequencing is comparable to use of MLST primers and fragment sequencing, it is appropriate to use more informative combinations of whole sequences. Future work will be toward implementing tests based on dCITE to indicate when a dataset lacks the necessary cladistic information from which to compute a robust, fully resolved tree. One possibility, suggested by Experiment 1, particularly Fig 2, is to compute something akin to rarefaction curves used by ecologists (see review [53]). That is, by looking at the cladistic information content, via dCITE scores, for samples of sequences one will be able to see whether cladistic information is showing signs of saturating.

## Supporting Information

S1 File The Supporting Information S1 File contains Figs A-C and Table A, supporting Experiment 1, Fig D supporting Experiment 2 and Figs E-G and Tables B-C supporting Experiment 3. (PDF)

## References

[1] Woese CR, Fox GE. Evolution Phylogenetic Structure of the Prokaryotic Domain: The Primary Kingdoms. Proc Natl Acad Sci USA. 1977;74:5088–5090. 10.1073/pnas.74.11.5088 270744 PMC432104

[2] Eisen JA. The recA Protein as a Model Molecule for Molecular Systematic Studies of Bacteria: Comparison of Trees of recAs and 16S rRNAs from the Same Species. J Mol Evol. 1995;41:1105–1123. 10.1007/BF00173192 8587109 PMC3188426

[3] Penny D, Hendy MD, Zimmer EA, Hamby K. Trees from Sequences: Panacea or Pandora’s Box? Aust Syst Bot. 1990;3:21–38. 10.1071/SB9900021

[4] D’Erchia AM, Gissi C, Pesole G, Saccone C, Arnason U. The Guinea-Pig is not a Rodent. Nature. 1996;381:597–600. 10.1038/381597a0 8637593

[5] Rokas A, Williams BL, King N, Carroll SB. Genome-Scale Approaches to Resolving Incongruence in Molecular Phylogenies. Nature. 2003;425:798–804. 10.1038/nature02053 14574403

[6] de Queiroz A, Gatesy J. The Supermatrix Approach to Systematics. Trends Ecol Evol. 2006;22:34–41. 10.1016/j.tree.2006.10.002 17046100

[7] Zhang J, Yang JR. Determinants of the Rate of Protein Sequence Evolution. Nat Rev Genet. 2015;16:409–420. 10.1038/nrg3950 26055156 PMC4523088

[8] Glenner H, Hansen AJ, Sørensen MV, Ronquist F, Huelsenbeck JP, Willerslev E. Bayesian Inference of the Metazoan Phylogeny: A Combined Molecular and Morphological Approach. Curr Biol. 2004;14:1644–1649. 10.1016/j.cub.2004.09.027 15380066

[9] Telford MJ, Wise MJ, Gowri-Shankar V. Consideration of RNA Secondary Structure Significantly Improves Likelihood-Based Estimates of Phylogeny: Examples from the Bilateria. Mol Biol Evol. 2005;22:1129–1136. 10.1093/molbev/msi099 15689526

[10] Koonin EV, Makarova KS, Aravind L. Horizontal Gene Transfer in Prokaryotes Quantification and Classification. Annu Rev Microbiol. 2001;55:709–742. 10.1146/annurev.micro.55.1.709 11544372 PMC4781227

[11] Keeling PJ, Palmer JD. Horizontal Gene Transfer in Eukaryotic Evolution. Nat Rev Genet. 2008;9:605–618. 10.1038/nrg2386 18591983

[12] Maddison WP. Gene Trees in Species Trees. Syst Biol. 1997;46:523–536. 10.1093/sysbio/46.3.523

[13] Doolittle WF. Phylogenetic Classification and the Universal Tree. Science. 1999;284:2124–2128. 10.1126/science.284.5423.2124 10381871

[14] Szöllősi GJ, Boussau B, Abby SS, Tannier E, Daubin V. Phylogenetic Modeling of Lateral Gene Transfer Reconstructs the Pattern and Relative Timing of Speciations. Proc Natl Acad Sci USA. 2012;109:17513–17518. 10.1073/pnas.1202997109 23043116 PMC3491530

[15] Jeffroy O, Brinkmann H, Delsuc F, Philippe H. Phylogenomics: The beginning of Incongruence? Trends Genet. 2006;22:225–231. 10.1016/j.tig.2006.02.003 16490279

[16] Strimmer K, von Haeseler A. Genetic Distances and Nucleotide Substitution Models—Theory In: Lemey P, Salemi M, Vandamme AM, editors. The Phylogenetic Handbook: a Practical Approach to Phylogenetic Analysis and Hypothesis Testing (2e). Cambridge University Press; 2009 p. 111–125.

[17] Pàl C, Papp B, Hurst LD. Highly Expressed Genes in Yeast Evolve Slowly. Genetics. 2001;158:927–931. 11430355 10.1093/genetics/158.2.927 PMC1461684

[18] Park C, Zhang J. High Expression Hampers Horizontal Gene Transfer. Genome Biol Evol. 2012;4:523–532. 10.1093/gbe/evs030 22436996 PMC3342876

[19] Dessimoz C, Gil M. Phylogenetic Assessment of Alignments Reveals Neglected Tree Signal in Gaps. Genome Biol. 2010;11:R37 10.1186/gb-2010-11-4-r37 20370897 PMC2884540

[20] Wise MJ. Mean Protein Evolutionary Distance: A Method for Comparative Protein Evolution and its Application. PLoS One. 2013;8:e61276 10.1371/journal.pone.0061276 23613826 PMC3626687

[21] Soltis DE, Albert VA, Savolainen V, Hilu K, Qiu YL, Chase MW, et al Genome-Scale Data, Angiosperm Relationships, and ‘Ending Incongruence’: A Cautionary Tale in Phylogenetics. Trends Plant Sci. 2004;9:477–483. 10.1016/j.tplants.2004.08.008 15465682

[22] Townsend JP, Lopez-Giraldez F. Optimal Selection of Gene and Ingroup Taxon Sampling for Resolving Phylogenetic Relationships. Syst Biol. 2010;59:446–457. 10.1093/sysbio/syq025 20547780

[23] Nabhan AR, Sarkar IN. The Impact of Taxon Sampling on Phylogenetic Inference: A Review of two Decades of Controversy. Brief Bioinform. 2012;13:122–134. 10.1093/bib/bbr014 21436145 PMC3251835

[24] Källersjö M, Albert VA, Farris JS. Homoplasy Increases Phylogenetic Structure. Cladistics. 1999;15:91–93. 10.1006/clad.1999.0085

[25] Nelson G, Platnick N. Systematic Patterns: Component Analysis In: Systematics and Biogeography: Cladistics and Vicariance. Columbia University Press; 1981 p. 169–265.

[26] Wortley AH, Scotland RW. Determining the Potential Utility of Datasets for Phylogeny Reconstruction. Taxon. 2006;55:431–442. 10.2307/25065589

[27] Maddison W. Reconstructing Character Evolution on Polytomous Cladograms. Cladistics. 1989;5:365–377. 10.1111/j.1096-0031.1989.tb00569.x 34933477

[28] Thorley JL, Wilkinson M, Charleston M. The Information Content of Consensus Trees. In: Rizzi A, Vichi M, Bock HH, editors. 6th Conference of the International Federation of Classification Societies (IFCS-98). Università *La Sapienza*, Rome; 1998. p. 91–97.

[29] Thorley JL. Cladistic Information, Leaf Stability and Supertree Construction. PhD Thesis, Department of Biological Sciences, University of Bristol; 2000.

[30] Massingham T, Goldman N. EDIBLE: Experimental Design and Information Calculations in Phylogenetics. Bioinformatics. 2000;16:294–295. 10.1093/bioinformatics/16.3.294 10869025

[31] San Mauro D, Gower DJ, Massingham T, Wilkinson M, Zardoya R, Cotton JA. Experimental Design in Caecilian Systematics: Phylogenetic Information of Mitochondrial Genomes and Nuclear *rag1*. Syst Biol. 2009;58:425–438. 10.1093/sysbio/syp043 20525595

[32] Cotton JA, Wilkinson M. Quantifying the Potential Utility of Phylogenetic Characters. Taxon. 2008;57:131–136.

[33] Steel M, Penny D. Maximum Parsimony and the Phylogenetic Information in Multistate Characters In: Albert VA, editor. Parsimony, Phylogeny, and Genomics. Oxford University Press; 2005 p. 163–178.

[34] The International HapMap Consortium. The International HapMap Project. Nature. 2003;426:789–796. 10.1038/nature02168 14685227

[35] Altschul SF, Gish W, Miller W, Myers EW, Lipman DJ. Basic Local Alignment Search Tool. J Mol Biol. 1990;215:403–410. 10.1016/S0022-2836(05)80360-2 2231712

[36] Edgar RC. Search and Clustering Orders of Magnitude Faster than BLAST. Bioinformatics. 2010;26:2460–2461. 10.1093/bioinformatics/btq461 20709691

[37] Edgar RC. MUSCLE: A Multiple Sequence Alignment Method with Reduced time and Space Complexity. BMC Bioinformatics. 2004;5(113). 10.1186/1471-2105-5-113 15318951 PMC517706

[38] Guindon S, Gascuel O. A Simple, Fast, and Accurate Algorithm to Estimate Large Phylogenies by Maximum Likelihood. Syst Biol. 2003;52:696–704. 10.1080/10635150390235520 14530136

[39] Felsenstein J. PHYLIP (Phylogeny Inference Package) version 3.7. Distributed by the author, Department of Genome Sciences, University of Washington, Seattle; 2009.

[40] Colless DH. Congruence Between Morphometric and Allozyme Data for *Menidia* Species: A Reappraisal. Syst Zool. 1980;29:288–299. 10.2307/2412663

[41] Ronquist F, Teslenko M, van der Mark P, Ayres DL, Darling A, Höhna S, et al MrBayes 3.2: Efficient Bayesian Phylogenetic Inference and Model Choice Across a Large Model Space. Syst Biol. 2012;61:539–542. 10.1093/sysbio/sys029 22357727 PMC3329765

[42] Spielman SJ, Wilke CO. Pyvolve: A Flexible Python Module for Simulating Sequences along Phylogenies. PLoS One. 2015;10:e0139047 10.1371/journal.pone.0139047 26397960 PMC4580465

[43] Bogdanowicz D, Giaro K, Wróbel B. TreeCmp: Comparison of Trees in Polynomial Time. Evol Bioinform. 2012;8:475–487. 10.4137/EBO.S9657

[44] Vandamme P, Dewhirst FE, Paster BJ, On SLW. Campylobacter In: Brenner DJ, Krieg NR, Staley JT, Garrity GM, editors. Bergey’s Manual of Systematic Bacteriology, Volume 2: The Proteobacteria. Springer; 2005 p. 1147–1160.

[45] Vandamme P, Dewhirst FE, Paster BJ, On SLW. Arcobacter In: Brenner DJ, Krieg NR, Staley JT, Garrity GM, editors. Bergey’s Manual of Systematic Bacteriology, Volume 2: The Proteobacteria. Springer; 2005 p. 1161–1164.

[46] On SLW, Lee A, O’Rourke JL, Dewhirst FE, Paster BJ, Fox JG, et al Helicobacter In: Brenner DJ, Krieg NR, Staley JT, Garrity GM, editors. Bergey’s Manual of Systematic Bacteriology, Volume 2: The Proteobacteria. Springer; 2005 p. 1169–1189.

[47] Kröger A, Klimmek O, Vandamme P, Dewhirst FE, Paster BJ. Wolinella In: Brenner DJ, Krieg NR, Staley JT, Garrity GM, editors. Bergey’s Manual of Systematic Bacteriology, Volume 2: The Proteobacteria. Springer; 2005 p. 1191–1194.

[48] Ishihama Y, Schmidt T, Rappsilber J, Mann M, Hart FU, Kerner MJ, et al Protein Abundance Profiling of the *Escherichia coli* Cytosol. BMC Genomics. 2008;9:102 10.1186/1471-2164-9-102 18304323 PMC2292177

[49] Maiden MCJ, Bygraves JA, Feil E, Morelli G, Russell JE, Urwin R, et al Multilocus Sequence Typing: A Portable Approach to the Identification of Clones Within Populations of Pathogenic Microorganisms. Proc Natl Acad Sci USA. 1998;95:3140–3145. 10.1073/pnas.95.6.3140 9501229 PMC19708

[50] Jolley KA, Maiden MCJ. BIGSdb: Scalable Analysis of Bacterial Genome Variation at the Population Level. BMC Bioinformatics. 2010;11:595 10.1186/1471-2105-11-595 21143983 PMC3004885

[51] Waterhouse AM, Procter JB, Martin DMA, Clamp M, Barton GJ. Jalview Version 2: A Multiple Sequence Alignment Editor and Analysis Workbench. Bioinformatics. 2009;25:1189–1191. 10.1093/bioinformatics/btp033 19151095 PMC2672624

[52] R Development Core Team. R: A Language and Environment for Statistical Computing; 2014.

[53] Gotelli NJ, Colwell RK. Estimating Species Richness In: Magurran AE, McGill BJ, editors. Biological Diversity: Frontiers in Measurement and Assessment. Oxford University Press; 2011 p. 39–54.

