# Supplementary material for: dCITE: Measuring Necessary Cladistic Information Can Help You Reduce Polytomy Artefacts in Trees
Source: PLoS One. 2016 Nov 29;11(11):e0166991. doi: 10.1371/journal.pone.0166991 (PMC5127522; doi:10.1371/journal.pone.0166991)
Supplement: S1 File — (PDF) [file pone.0166991.s001.pdf]

Supporting Information S1 for: dCITE: Measuring Necessary Cladistic Information can help you Reduce Polytomy Artefacts in Trees

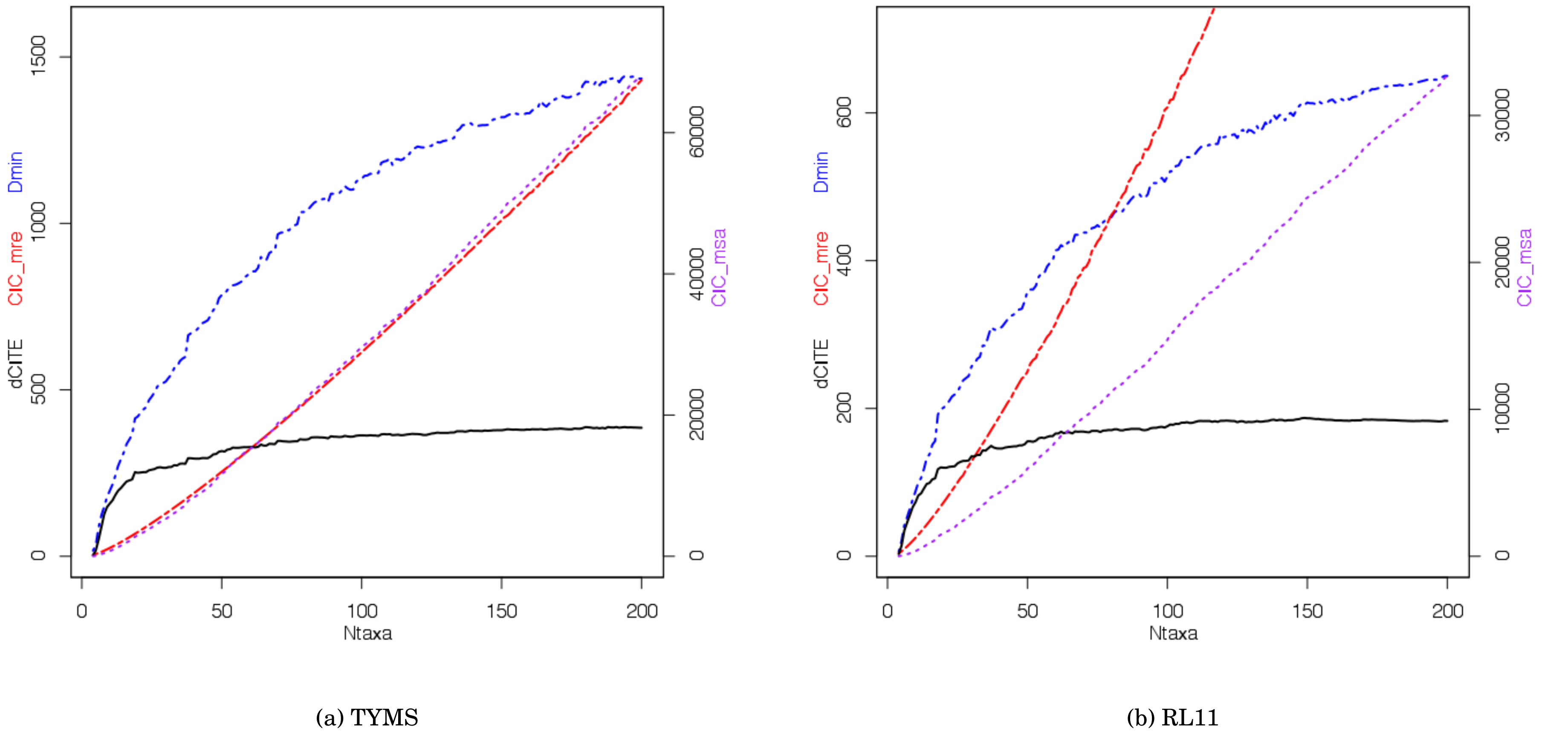

Figure A. Experiment 1. For the TYMS-98 dataset (a) and the RL11-98 dataset (b), the plot of dCITE score (solid black line), $\Delta_m in$ (shown as a blue . – . – line), computed from the input multiple sequence alignments, and CIC scores based on the MRE trees (shown in red using a . — . — line) are plotted versus the counts of taxa. TOTAL CIC scores based on the input multiple sequence alignments, also plotted against counts of taxa, are shown using purple dashed lines. Note the different Y-axis for these scores, on the right hand side.

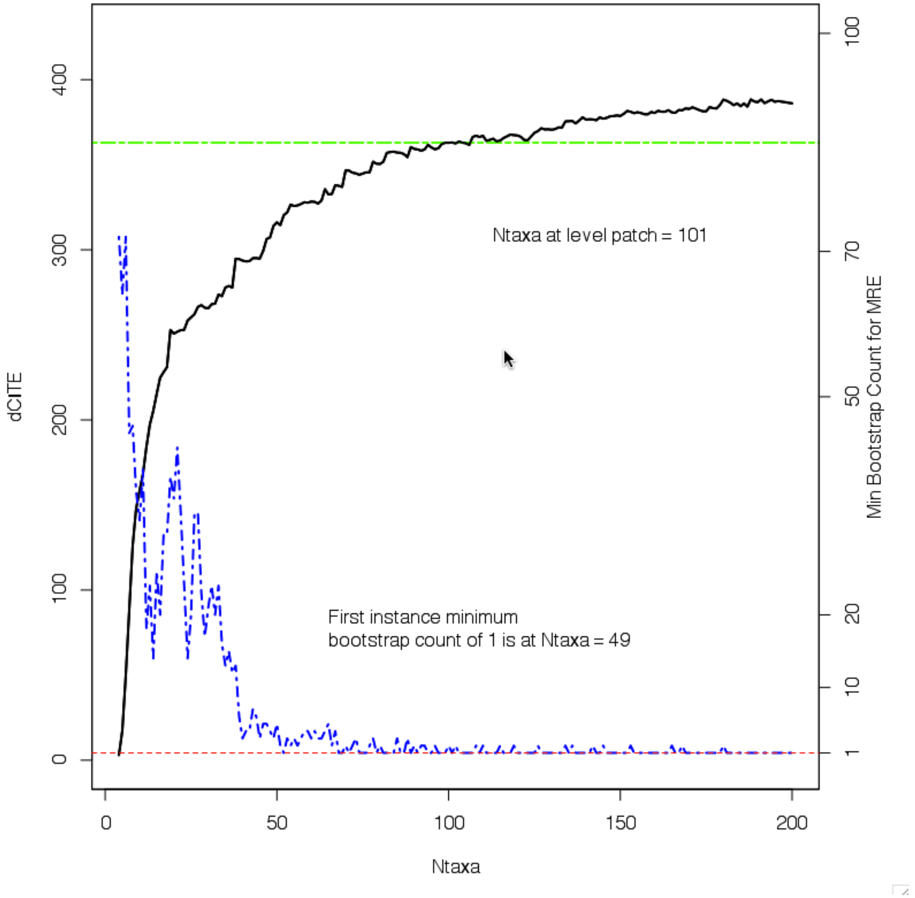

(a) TYMS

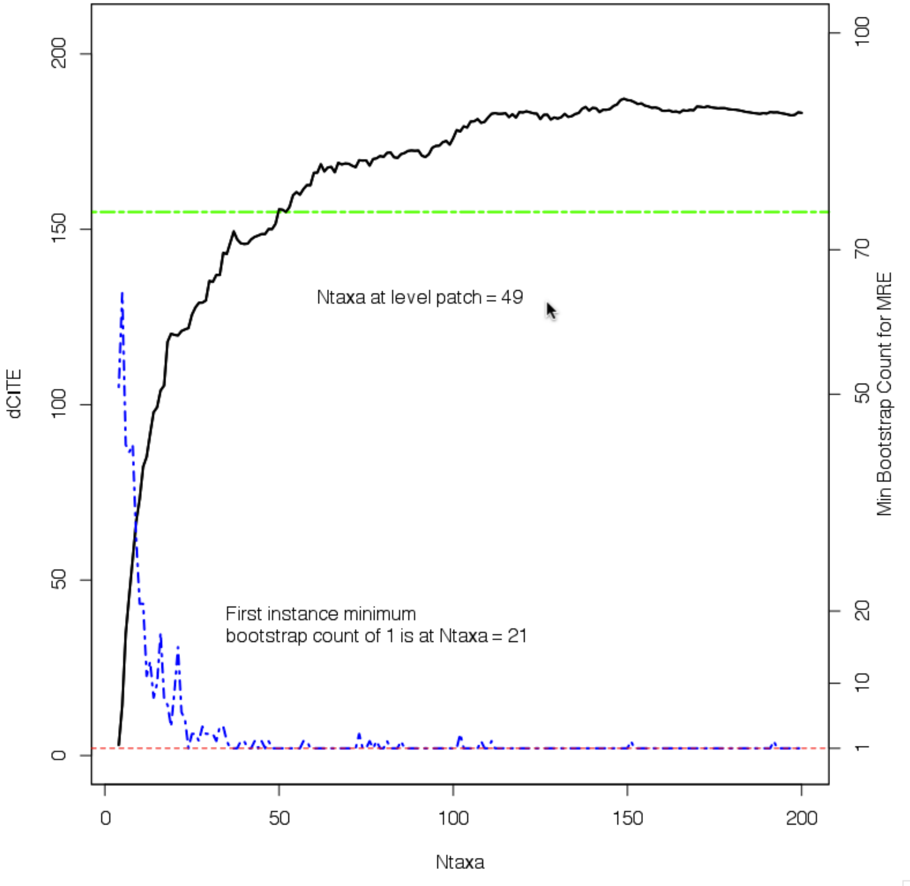

(b) RL11

Figure B. Experiment 1. For the TYMS-98 dataset(a) and the RL11-98 dataset (b), the plot of dCITE score (black solid line) and Minimum Bootstrap Count (for trees created using the Majority Rule Extended consensus rule, shown as a blue . – . – line) versus the counts of taxa. A horizontal green line marks the first "level patch", i.e. where the sum of the differences between adjacent dCITE scores over 15 counts of taxa is no more than 15.

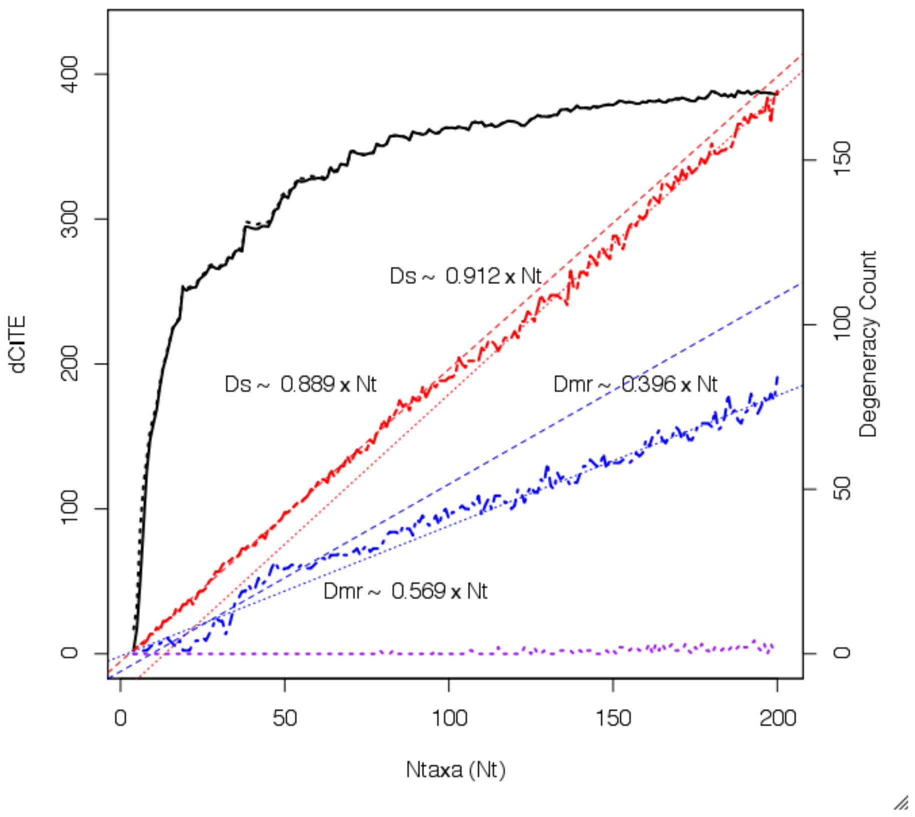

(a) TYMS

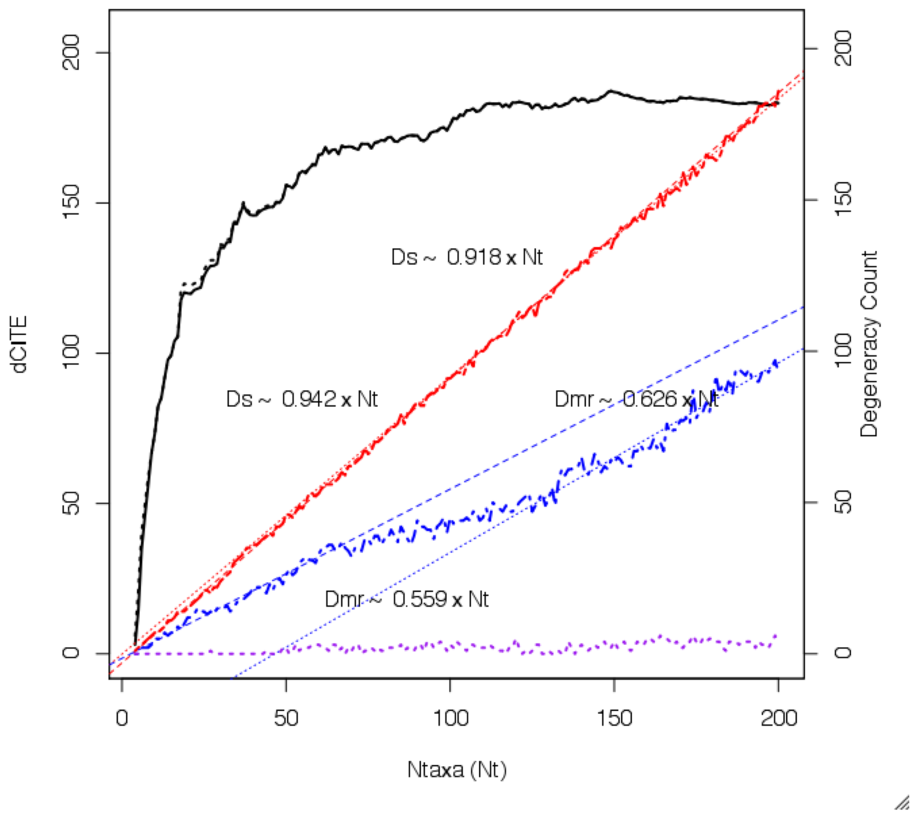

(b) RL11

Figure C. Experiment 1. For the TYMS-98 dataset(a) and the RL11-98 dataset (b), the plot of dCITE score (solid black line) plotted against counts of taxa. Scores are on the left hand side Y axis. The dashed black line next to the dCITE score graph is the graph of the corresponding CITE scores. Based on the Y axis on the right hand side there are the plots of degeneracy scores for Strict consensus trees (shown in red in red using a . — . — line), Majority Rule consensus trees (shown as a blue . – . – line) and Majority Rule Extended consensus trees (shown shown as a - - - - purple line at the bottom of the graph). For the Strict consensus trees and the MR trees linear models (dashed red and blue lines, respectively) are also shown. For each of the linear models, the slope Degeneracy (D) with the counts of taxa (Ntaxa) is also noted.

Table A. Experiment 1. Mean, median and max character diversity scores, and the fraction of scores less than or equal to 0.5, for the ATPA dataset for N = 4 to 200 taxa

N	Mean	Median	Max	Sites	LE0.5	N	Mean	Median	Max	Sites	LE0.5	N	Mean	Median	Max	Sites	LE0.5
4	0.500	0.500	0.500	32	1.000	5	0.402	0.400	0.500	89	1.000	6	0.342	0.333	0.500	150	1.000
7	0.304	0.286	0.500	187	1.000	8	0.279	0.250	0.500	217	1.000	9	0.258	0.222	0.444	242	1.000
10	0.243	0.200	0.400	271	1.000	11	0.231	0.182	0.455	275	1.000	12	0.218	0.167	0.417	292	1.000
13	0.209	0.231	0.385	301	1.000	14	0.199	0.214	0.429	308	1.000	15	0.191	0.200	0.400	311	1.000
16	0.184	0.188	0.375	316	1.000	17	0.181	0.176	0.500	329	1.000	18	0.174	0.167	0.333	340	1.000
19	0.167	0.158	0.333	341	1.000	20	0.162	0.150	0.350	343	1.000	21	0.167	0.150	0.400	354	1.000
22	0.170	0.150	0.400	360	1.000	23	0.171	0.150	0.450	360	1.000	24	0.173	0.150	0.450	363	1.000
25	0.177	0.150	0.450	366	1.000	26	0.180	0.150	0.450	366	1.000	27	0.183	0.150	0.500	369	1.000
28	0.186	0.150	0.500	372	1.000	29	0.187	0.150	0.500	373	1.000	30	0.189	0.200	0.500	373	1.000
31	0.195	0.200	0.500	376	1.000	32	0.197	0.200	0.500	377	1.000	33	0.199	0.200	0.550	378	0.997
34	0.201	0.200	0.550	378	0.997	35	0.204	0.200	0.600	378	0.995	36	0.207	0.200	0.600	379	0.995
37	0.207	0.200	0.600	381	0.995	38	0.208	0.200	0.600	382	0.995	39	0.213	0.200	0.600	385	0.995
40	0.215	0.200	0.600	386	0.995	41	0.216	0.200	0.600	386	0.995	42	0.218	0.200	0.600	387	0.987
43	0.219	0.200	0.600	388	0.985	44	0.220	0.200	0.600	388	0.985	45	0.223	0.200	0.600	388	0.982
46	0.223	0.200	0.600	389	0.982	47	0.225	0.200	0.600	390	0.982	48	0.229	0.200	0.600	391	0.982
49	0.230	0.200	0.600	392	0.982	50	0.231	0.200	0.600	391	0.982	51	0.234	0.200	0.600	392	0.985
52	0.235	0.200	0.600	393	0.982	53	0.235	0.200	0.600	395	0.982	54	0.236	0.200	0.600	397	0.977
55	0.237	0.200	0.600	397	0.977	56	0.238	0.200	0.650	398	0.977	57	0.238	0.200	0.650	401	0.978
58	0.239	0.200	0.650	400	0.978	59	0.240	0.200	0.650	401	0.975	60	0.240	0.200	0.650	400	0.975
61	0.242	0.200	0.650	401	0.970	62	0.242	0.200	0.650	402	0.968	63	0.242	0.200	0.650	402	0.970
64	0.242	0.200	0.650	403	0.970	65	0.243	0.200	0.650	404	0.970	66	0.244	0.200	0.650	404	0.973
67	0.246	0.200	0.650	406	0.966	68	0.246	0.200	0.650	406	0.966	69	0.250	0.200	0.650	407	0.963
70	0.251	0.250	0.650	407	0.961	71	0.252	0.250	0.650	408	0.958	72	0.252	0.250	0.650	409	0.956
73	0.253	0.250	0.650	409	0.954	74	0.254	0.250	0.650	409	0.954	75	0.254	0.250	0.650	409	0.956

N	Mean	Median	Max	Sites	LE0.5	N	Mean	Median	Max	Sites	LE0.5	N	Mean	Median	Max	Sites	LE0.5
76	0.255	0.250	0.650	409	0.956	77	0.256	0.250	0.650	409	0.956	78	0.257	0.250	0.650	409	0.956
79	0.258	0.250	0.650	409	0.954	80	0.258	0.250	0.650	409	0.954	81	0.259	0.250	0.650	410	0.951
82	0.259	0.250	0.650	410	0.954	83	0.260	0.250	0.650	410	0.954	84	0.262	0.250	0.650	414	0.954
85	0.262	0.250	0.650	414	0.954	86	0.262	0.250	0.650	414	0.954	87	0.263	0.250	0.650	414	0.949
88	0.263	0.250	0.650	414	0.949	89	0.263	0.250	0.650	415	0.947	90	0.263	0.250	0.650	414	0.947
91	0.263	0.250	0.650	415	0.947	92	0.264	0.250	0.650	415	0.947	93	0.264	0.250	0.650	415	0.947
94	0.266	0.250	0.650	415	0.947	95	0.266	0.250	0.650	416	0.947	96	0.268	0.250	0.650	416	0.942
97	0.269	0.250	0.700	416	0.940	98	0.269	0.250	0.700	416	0.935	99	0.269	0.250	0.700	416	0.935
100	0.271	0.250	0.700	416	0.933	101	0.270	0.250	0.700	416	0.933	102	0.271	0.250	0.700	416	0.930
103	0.271	0.250	0.700	416	0.928	104	0.271	0.250	0.700	416	0.928	105	0.272	0.250	0.700	416	0.928
106	0.272	0.250	0.750	415	0.925	107	0.273	0.250	0.700	416	0.923	108	0.273	0.250	0.750	415	0.930
109	0.274	0.250	0.750	415	0.930	110	0.275	0.250	0.750	415	0.930	111	0.275	0.250	0.750	415	0.930
112	0.276	0.250	0.750	415	0.930	113	0.275	0.250	0.750	415	0.928	114	0.276	0.250	0.750	418	0.919
115	0.276	0.250	0.750	419	0.921	116	0.277	0.250	0.750	419	0.919	117	0.278	0.250	0.750	419	0.919
118	0.279	0.250	0.750	419	0.916	119	0.279	0.250	0.750	419	0.916	120	0.279	0.250	0.750	419	0.914
121	0.280	0.250	0.750	419	0.909	122	0.284	0.250	0.750	419	0.905	123	0.288	0.250	0.750	419	0.902
124	0.289	0.250	0.750	419	0.888	125	0.289	0.250	0.750	419	0.888	126	0.290	0.250	0.750	419	0.890
127	0.290	0.250	0.800	420	0.888	128	0.291	0.250	0.800	420	0.895	129	0.291	0.250	0.800	421	0.891
130	0.292	0.250	0.800	421	0.886	131	0.294	0.250	0.800	421	0.891	132	0.294	0.250	0.800	421	0.891
133	0.294	0.250	0.800	420	0.895	134	0.294	0.250	0.800	420	0.890	135	0.295	0.250	0.800	422	0.891
136	0.295	0.250	0.800	422	0.893	137	0.296	0.250	0.800	422	0.886	138	0.297	0.250	0.800	423	0.891
139	0.296	0.250	0.800	423	0.891	140	0.297	0.250	0.800	423	0.891	141	0.298	0.250	0.800	423	0.889
142	0.298	0.250	0.800	423	0.884	143	0.298	0.250	0.800	423	0.889	144	0.300	0.250	0.800	421	0.888
145	0.300	0.250	0.800	422	0.889	146	0.300	0.250	0.800	423	0.891	147	0.300	0.250	0.800	423	0.882
148	0.301	0.250	0.800	423	0.891	149	0.301	0.250	0.800	423	0.891	150	0.301	0.250	0.800	423	0.887
151	0.302	0.250	0.800	423	0.889	152	0.303	0.250	0.800	422	0.889	153	0.304	0.250	0.800	423	0.877

N	Mean	Median	Max	Sites	LE0.5	N	Mean	Median	Max	Sites	LE0.5	N	Mean	Median	Max	Sites	LE0.5
154	0.305	0.250	0.800	424	0.875	155	0.305	0.250	0.800	424	0.875	156	0.305	0.250	0.800	424	0.877
157	0.305	0.250	0.800	424	0.880	158	0.306	0.250	0.800	424	0.880	159	0.307	0.250	0.800	424	0.877
160	0.308	0.250	0.800	425	0.873	161	0.308	0.250	0.800	426	0.866	162	0.309	0.250	0.800	426	0.862
163	0.309	0.250	0.800	426	0.862	164	0.309	0.250	0.800	425	0.861	165	0.309	0.250	0.800	425	0.861
166	0.310	0.250	0.800	425	0.861	167	0.310	0.250	0.800	425	0.856	168	0.310	0.250	0.800	425	0.856
169	0.311	0.250	0.800	426	0.857	170	0.311	0.250	0.800	426	0.857	171	0.311	0.250	0.800	427	0.857
172	0.310	0.250	0.800	427	0.855	173	0.312	0.250	0.800	427	0.852	174	0.312	0.250	0.800	426	0.852
175	0.313	0.250	0.800	427	0.848	176	0.314	0.250	0.800	427	0.841	177	0.314	0.250	0.800	427	0.838
178	0.314	0.250	0.800	427	0.838	179	0.313	0.250	0.800	427	0.843	180	0.313	0.250	0.800	427	0.843
181	0.314	0.250	0.800	427	0.843	182	0.314	0.250	0.800	427	0.843	183	0.314	0.250	0.800	428	0.841
184	0.315	0.250	0.800	428	0.839	185	0.315	0.250	0.800	428	0.836	186	0.316	0.250	0.800	428	0.839
187	0.316	0.250	0.800	428	0.839	188	0.317	0.250	0.800	427	0.836	189	0.317	0.300	0.800	427	0.831
190	0.317	0.275	0.800	426	0.833	191	0.319	0.300	0.800	428	0.829	192	0.318	0.300	0.800	428	0.832
193	0.320	0.300	0.800	429	0.830	194	0.321	0.300	0.800	429	0.825	195	0.320	0.300	0.800	427	0.824
196	0.320	0.300	0.800	427	0.824	197	0.321	0.300	0.800	428	0.827	198	0.321	0.300	0.800	427	0.827
199	0.322	0.300	0.800	428	0.820	200	0.324	0.300	0.800	429	0.818						

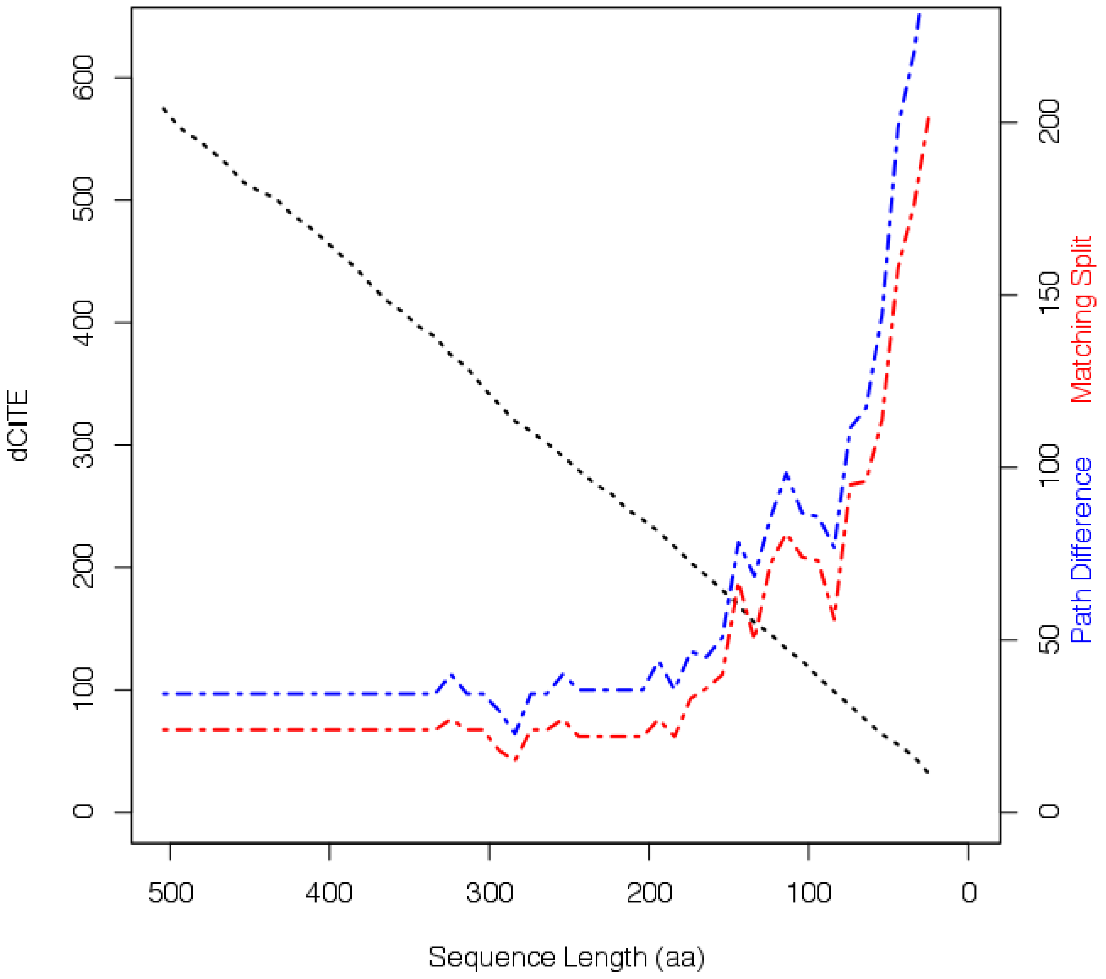

Figure D. Experiment 2. dCITE score for input multiple sequence alignment, and Matching Split and Path Difference scores for computed consensus tree versus the True Tree, plotted against sequence length. For the 50 taxon ATPA-98 dataset, the plot of dCITE score (solid black line) plotted against length of the input multiple sequence alignment. Also plotted against the length of the multiple sequence alignment are the Path Difference (blue .— line) and Matching Split (red .— line) scores for the difference between the Majority Rule Extended consensus trees and the True Tree.

Table B. List of species examined for Experiment 3

Arcobacter butzleri 7h1h	Arcobacter butzleri RM4018	Arcobacter nitrofigilis ATCC 33309
Campylobacter coli JV20	Campylobacter concisus 13826	Campylobacter curvus 525.92
Campylobacter fetus fetus 82 40	Campylobacter gracilis RM3268	Campylobacter jejuni 1336
Campylobacter jejuni 260.94	Campylobacter jejuni 305	Campylobacter jejuni 327
Campylobacter jejuni 414	Campylobacter jejuni CF93 6	Campylobacter jejuni CG8486
Campylobacter jejuni DFVF1099	Campylobacter jejuni HB93 13	Campylobacter jejuni HS21 M1
Campylobacter jejuni HS41 ICDCCJ07001	Campylobacter jejuni IA3902	Campylobacter jejuni NCTC 11168
Campylobacter jejuni O23	Campylobacter jejuni O6 81116	Campylobacter jejuni RM1221
Campylobacter jejuni S3	Campylobacter lari RM2100	Campylobacter rectus RM3267
Campylobacter showae RM3277	Campylobacter upsaliensis JV21	Campylobacter upsaliensis RM3195
Helicobacter acinonychis Sheeba	Helicobacter bizzozeronii CCUG 35545	Helicobacter canadensis MIT 98 5491
Helicobacter cetorum MIT 00 7128	Helicobacter cinaedi PAGU611	Helicobacter felis ATCC 49179
Helicobacter fennelliae MRY12 0050	Helicobacter heilmannii ASB1.4	Helicobacter hepaticus
Helicobacter mustelae ATCC 43772	Helicobacter pullorum MIT 98 5489	Helicobacter pylori 26695
Helicobacter pylori 35A	Helicobacter pylori 51	Helicobacter pylori 52
Helicobacter pylori 908	Helicobacter pylori 98 10	Helicobacter pylori Aklavik117
Helicobacter pylori B128	Helicobacter pylori B38	Helicobacter pylori B8
Helicobacter pylori Cuz20	Helicobacter pylori ELS37	Helicobacter pylori F16
Helicobacter pylori F30	Helicobacter pylori F32	Helicobacter pylori F57
Helicobacter pylori G27	Helicobacter pylori Gambia94	Helicobacter pylori HPAG1
Helicobacter pylori India7	Helicobacter pylori J99	Helicobacter pylori Lithuania75
Helicobacter pylori NCTC 11637	Helicobacter pylori P12	Helicobacter pylori PeCan4
Helicobacter pylori Puno135	Helicobacter pylori SJM180	Helicobacter pylori SNT49
Helicobacter pylori Sahul64	Helicobacter pylori Sat464	Helicobacter pylori Shi470
Helicobacter pylori SouthAfrica7	Helicobacter pylori v225d	Helicobacter suis HS1
Helicobacter suis HS5	Helicobacter winghamensis ATCC BAA 430	Wolinella succinogenes

Table C. List of Helicobacter/Campylobacter Protein Identifiers and Corresponding *E. coli* Identifiers, with their UniProt Identifiers and Descriptions, for Experiment 3

Hp ID	HpUniProt	Ec ID	Ec UniProt	Description
accB	Q9ZKC9_HELPJ	accB	BCCP_ECOLI	Biotin carboxyl carrier protein of acetyl-CoA carboxylase
adk	KAD_HELPY	adk	KAD_ECOLI	Adenylate kinase (EC 2.7.4.3) (ATP-AMP transphosphorylase)
alaS	SYA_HELPY	alaS	SYA_ECOLI	Alanyl-tRNA synthetase (EC 6.1.1.7) (Alanine–tRNA ligase)
asd	DHAS_HELPY	asd	DHAS_ECOLI	Aspartate-semialdehyde dehydrogenase (EC 1.2.1.11)
aspA	ASPA_HELPY	aspA	ASPA_ECOLI	Aspartate ammonia-lyase (EC 4.3.1.1) (Aspartase)
atpA	ATPA_HELPY	atpA	ATPA_ECOLI	ATP synthase alpha chain (EC 3.6.3.14)
atpF	ATPF_HELPY	atpF	ATPF_ECOLI	ATP synthase B chain (EC 3.6.3.14)
atpG	ATPG_HELPY	atpG	ATPG_ECOLI	ATP synthase gamma chain (EC 3.6.3.14)
clpP	CLPP_HELPY	clpP	CLPP_ECOLI	ATP-dependent Clp protease proteolytic subunit (EC 3.4.21.92)
clpX	CLPX_HELPY	clpX	CLPX_ECOLI	ATP-dependent Clp protease ATP-binding subunit clpX
cysE	CYSE_HELPY	cysE	CYSE_ECOLI	Serine acetyltransferase (EC 2.3.1.30) (SAT)
dapA	DAPA_HELPY	dapA	DAPA_ECOLI	Dihydrodipicolinate synthase (EC 4.2.1.52) (DHDPS)
ddl	DDL_HELPY	ddlA	DDLA_ECOLI	D-alanine–D-alanine ligase A (EC 6.3.2.4)
def	DEF_HELPY	def	DEF_ECOLI	Peptide deformylase (EC 3.5.1.88) (PDF) (Polypeptide deformylase)
dnaE	DPO3A_HELPY	dnaE	DP3A_ECOLI	DNA polymerase III alpha subunit (EC 2.7.7.7)
dnaJ	DNAJ_HELPY	dnaJ	DNAJ_ECOLI	Chaperone protein dnaJ (Heat shock protein J) (HSP40)
dnaK	DNAK_HELPY	dnaK	DNAK_ECOLI	Chaperone protein dnaK (Heat shock protein 70)
efp	EFP_HELPY	efp	EFP_ECOLI	Elongation factor P (EF-P)
engA	DER_HELPY	der	DER_ECOLI	GTP-binding protein engA (Double era-like domain protein)
eno	ENO_HELPY	eno	ENO_ECOLI	Enolase (EC 4.2.1.11) (2-phosphoglycerate dehydratase)
fabD	O24916_HELPY	fabD	FABD_ECOLI	Malonyl CoA-acyl carrier protein transacylase (EC 2.3.1.39)
fabH	FABH_HELPY	fabH	FABH_ECOLI	3-oxoacyl-[acyl-carrier-protein] synthase III (EC 2.3.1.41)

fba	ALF_HELPY	fbaA	ALF_ECOLI	Fructose-bisphosphate aldolase class II (EC 4.1.2.13)
fbp	F16PA_HELPY	fbp	F16PA_ECOLI	Fructose-1,6-bisphosphatase
ffh	SRP54_HELPY	ffh	SRP54_ECOLI	Signal recognition particle protein (Fifty-four homolog)
fldA	FLAV_HELPY	fldA	FLAV_ECOLI	Flavodoxin 1
frdA	FRDA_HELPY	frdA	FRDA_ECOLI	Fumarate reductase flavoprotein subunit (EC 1.3.99.1)
ftnA	FTN_HELPY	ftnA	FTNA_ECOLI	Non heme iron-containing ferritin
ftsY	FTSY_HELPY	ftsY	FTSY_ECOLI	Cell division protein ftsY
ftsZ	FTSZ_HELPY	ftsZ	FTSZ_ECOLI	Cell division protein ftsZ
fur	FUR_HELPY	fur	FUR_ECOLI	Ferric uptake regulator
fusA	EFG_HELPY	fusA	EFG_ECOLI	Elongation factor G (EF-G)
glyQ	SYGA_HELPY	glyQ	SYGA_ECOLI	Glycyl-tRNA synthetase alpha chain (EC 6.1.1.14)
glyS	SYGB_HELPY	glyS	SYGB_ECOLI	Glycyl-tRNA synthetase beta chain (EC 6.1.1.14)
gmhB	GMHB_HELPY	gmhB	GMHB_ECOLI	D,D-heptose 1,7-bisphosphate phosphatase
gmk	KGUA_HELPY	gmk	KGUA_ECOLI	Guanylate kinase (EC 2.7.4.8) (GMP kinase)
gpsA	GPDA_HELPY	gpsA	GPDA_ECOLI	Glycerol-3-phosphate dehydrogenase [NAD(P)+] (EC 1.1.1.94)
groL	CH60_HELPY	groL	CH60_ECOLI	60 kDa chaperonin (Protein Cpn60) (groEL protein)
grpE	GRPE_HELPY	grpE	GRPE_ECOLI	GrpE protein (HSP-70 cofactor) (HSP24)
hisS	SYH_HELPY	hisS	SYH_ECOLI	Histidyl-tRNA synthetase (EC 6.1.1.21)
hslV	HSLV_HELPY	hslV	HSLV_ECOLI	ATP-dependent protease hslV (EC 3.4.25.-) (Heat shock protein hslV)
ileS	SYI_HELPY	ileS	SYI_ECOLI	Isoleucyl-tRNA synthetase (EC 6.1.1.5)
iscS	ISCS_HELPY	iscS	ISCS_ECOLI	Cysteine desulfurase (EC 2.8.1.7) (NifS protein homolog)
leuS	SYL_HELPY	leuS	SYL_ECOLI	Leucyl-tRNA synthetase (EC 6.1.1.4) (LeuRS)
lolA	LOLA_HELPY	lolA	LOLA_ECOLI	Outer-membrane lipoprotein carrier protein precursor (P20)
lon	LON_HELPY	lon	LON_ECOLI	ATP-dependent protease La (EC 3.4.21.53)
lpxD	LPXD_HELPY	lpxD	LPXD_ECOLI	UDP-3-O-[3-hydroxymyristoyl] glucosamine N-acyltransferase (EC 2.3.1.-)
mutY	O24954_HELPY			A/G-specific adenine glycosylase
nuoA	O25850_HELPY	nuoA	NUOA_ECOLI	NADH-quinone oxidoreductase chain A (EC 1.6.99.5)

nuoB	NUOB_HELPY	nuoB	NUOB_ECOLI	NADH-quinone oxidoreductase chain B (EC 1.6.99.5)
nuoD	NUOD_HELPY	nuoCD	NUCD_ECOLI	NADH-quinone oxidoreductase chain C/D (EC 1.6.99.5)
nusA	NUSA_HELPY	nusA	NUSA_ECOLI	Transcription elongation protein nusA (L factor)
nusB	NUSB_HELPY	nusB	NUSB_ECOLI	N utilization substance protein B (NusB protein)
nusG	NUSG_HELPY	nusG	NUSG_ECOLI	Transcription antitermination protein nusG
pgi	G6PI_HELPY	pgi	G6PI_ECOLI	Glucose-6-phosphate isomerase (EC 5.3.1.9) (GPI)
pgk	PGK_HELPY	pgk	PGK_ECOLI	Phosphoglycerate kinase (EC 2.7.2.3)
pheT	SYFB_HELPY	pheT	SYFB_ECOLI	Phenylalanyl-tRNA synthetase beta chain (EC 6.1.1.20)
pnp	PNP_HELPY	pnp	PNP_ECOLI	Polyribonucleotide nucleotidyltransferase (EC 2.7.7.8)
ppa	IPYR_HELPY	ppa	IPYR_ECOLI	Inorganic pyrophosphatase (EC 3.6.1.1)
prfB	RF2_HELPY	prfB	RF2_ECOLI	Peptide chain release factor 2 (RF-2)
purA	PURA_HELPY	purA	PURA_ECOLI	Adenylosuccinate synthetase (EC 6.3.4.4) (IMP–aspartate ligase)
pyrH	PYRH_HELPY	pyrH	PYRH_ECOLI	Uridylate kinase (EC 2.7.4.-) (UK) (Uridine monophosphate kinase)
recA	RECA_HELPY	recA	RECA_ECOLI	RecA protein (Recombinase A)
rho	RHO_HELPY	rho	RHO_ECOLI	Transcription termination factor rho
ribH	RISB_HELPY	ribE	RISB_ECOLI	6,7-dimethyl-8-ribityllumazine synthase
rimM	RIMM_HELPY	rimM	RIMM_ECOLI	16S rRNA processing protein rimM (21K)
rplD	RL4_HELPY	rplD	RL4_ECOLI	50S ribosomal protein L4
rplP	RL16_HELPY	rplP	RL16_ECOLI	50S ribosomal protein L16
rpoA	RPOA_HELPY	rpoA	RPOA_ECOLI	DNA-directed RNA polymerase alpha chain (EC 2.7.7.6)
rpoB	RPOB_ECOLI	rpoB	RPOB_ECOLI	DNA-directed RNA polymerase beta chain (EC 2.7.7.6)
rpoD	RPOD_HELPY	rpoD	RPOD_ECOLI	RNA polymerase sigma factor rpoD (Sigma-70)
rpsA	RS1_HELPY	rpsA	RS1_ECOLI	30S ribosomal protein S1
secY	SECY_HELPY	secY	SECY_ECOLI	Preprotein translocase secY subunit
slyD	SLYD_HELPY	slyD	SLYD_ECOLI	FKBP-type peptidyl-prolyl cis-trans isomerase slyD (EC 5.2.1.8)
sodB	SODF_HELPY	sodB	SODF_ECOLI	Superoxide dismutase [Fe] (EC 1.15.1.1)
tal	TAL_HELPY	talA	TALA_ECOLI	Transaldolase A (EC 2.2.1.2)

tatB	TATB_HELPY	tatB	TATB_ECOLI	Sec-independent protein translocase protein
thrS	SYT_HELPY	thrS	SYT_ECOLI	Threonyl-tRNA synthetase (EC 6.1.1.3)
tig	TIG_HELPY	tig	TIG_ECOLI	Trigger factor (TF)
tolB	TOLB_HELPY	tolB	TOLB_ECOLI	TolB protein precursor
tpiA	TPIS_HELPY	tpiA	TPIS_ECOLI	Triosephosphate isomerase (EC 5.3.1.1)
trmD	TRMD_HELPY	trmD	TRMD_ECOLI	tRNA (Guanine-N(1)-)-methyltransferase (EC 2.1.1.31)
trpC	TRPC_HELPY			Bifunctional indole-3-glycerol phosphate synthase/phosphoribosylanthranilate isomerase
truD	TRUD_HELPY	truD	TRUD_ECOLI	tRNA pseudouridine synthase D (EC 4.2.1.70)
tsf	EFTS_HELPY	tsf	EFTS_ECOLI	Elongation factor Ts (EF-Ts)

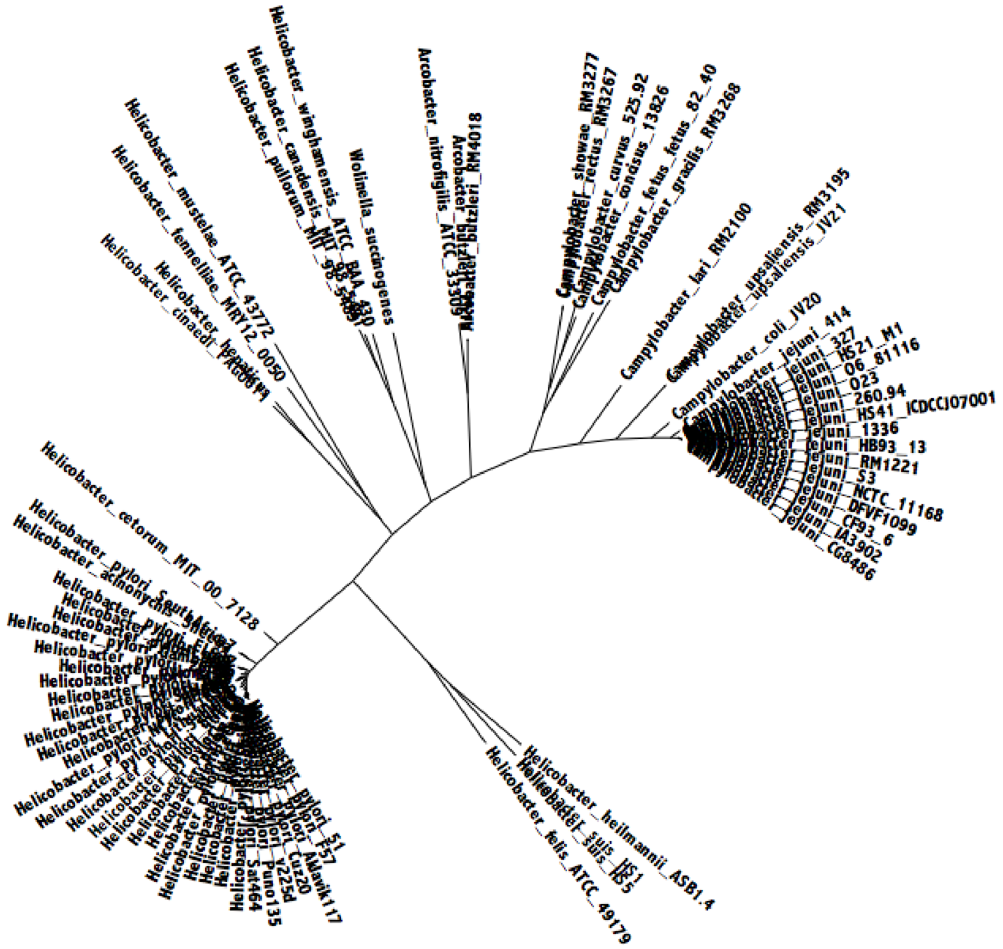

Figure E. Experiment 3. The Majority Rule phylogenetic tree of species from the order Campylobacterales constructed by the application MrBayes using a partitioned analysis based on the proteins glyS and pheT. The tree has been visualised using FigTree.

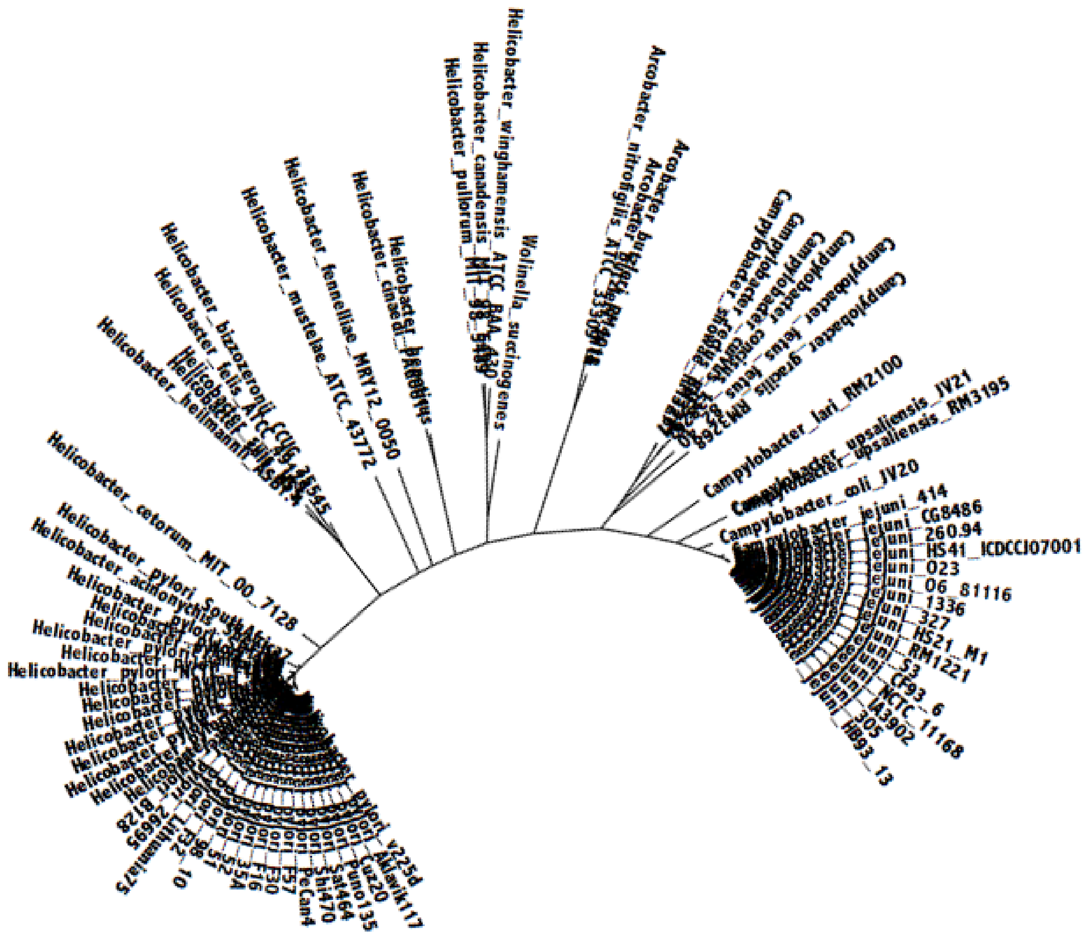

Figure F. Experiment 3. The Majority Rule phylogenetic tree of species from the order Campylobacterales constructed by the application MrBayes using a partitioned analysis based on the proteins lon and rpsA. The tree has been visualised using FigTree.

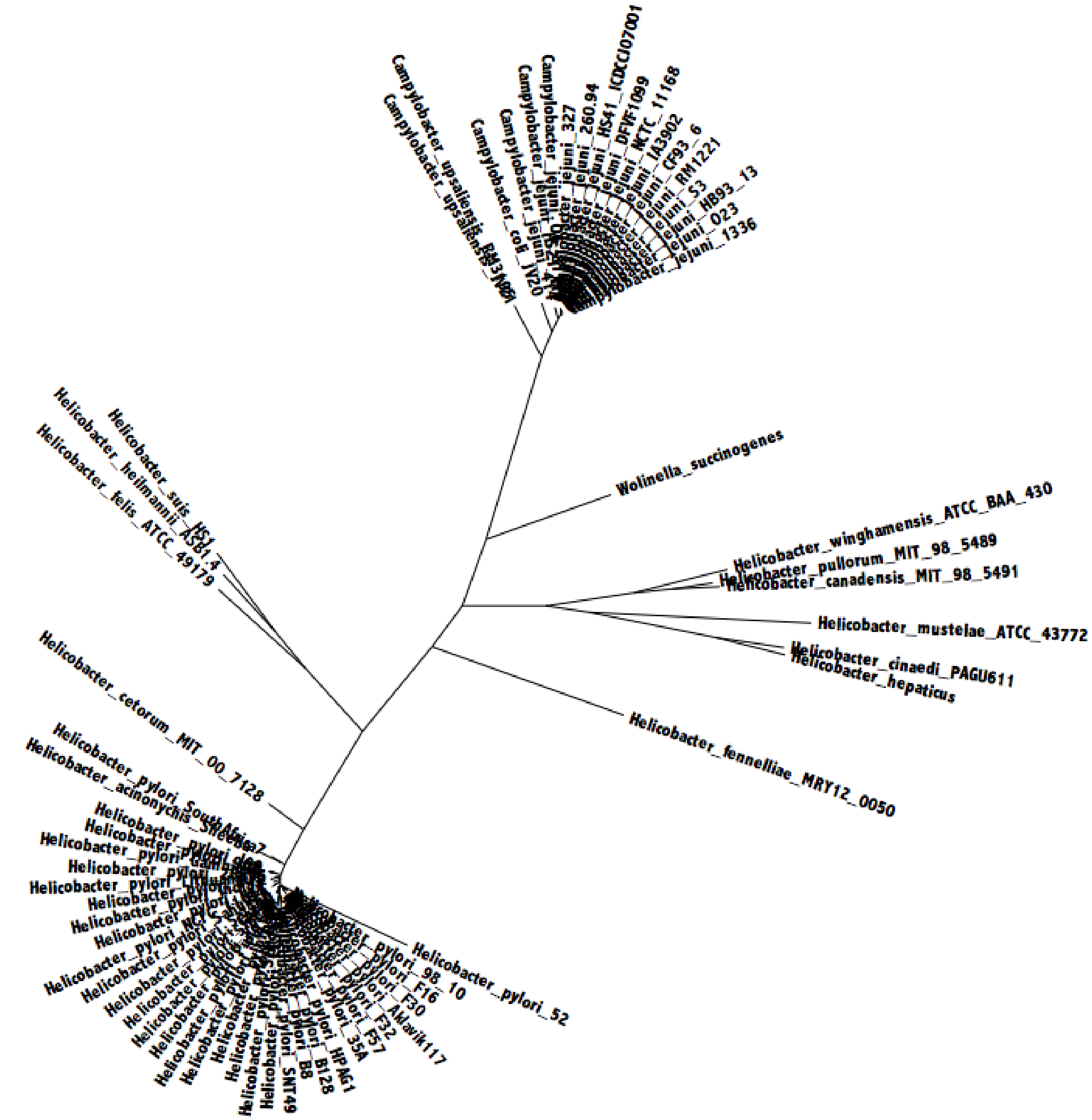

Figure G. Experiment 3. The Majority Rule phylogenetic tree of species from the order Campylobacterales constructed by the application MrBayes using a partitioned analysis based on the protein fragments corresponding to those from atpA, efp, mutY, trpC and engA used in MLST analyses. The tree has been visualised using FigTree.
